# An Experimental Study on Fiber Reinforcement of a Polymer TSL Material

**DOI:** 10.3390/polym18161992

**Published:** 2026-08-15

**Authors:** Han Liang, Daisong Liu, Yunjing Shi, Zihan Bai, Kangdong Shi, Chen Cao, Zedi Zhang

**Affiliations:** College of Mining, Liaoning Technical University, Fuxin 123000, China; 18641822228@163.com (H.L.); 18636593388@163.com (Y.S.); 13637172416@163.com (Z.B.); s1221hunter@163.com (K.S.); caochen@lntu.edu.cn (C.C.); 13203861312@163.com (Z.Z.)

**Keywords:** thin spray-on liner, fiber reinforcement, polyurea-silicate-based TSL, polyvinyl alcohol fiber, toughened polypropylene fiber, buffered shear test, true triaxial test, energy absorption

## Abstract

Thin spray-on liner (TSL) technology provides rapid and highly automated surface support for underground coal mine roadways. However, in deep roadways affected by high in situ stress, mining-induced disturbances, and fractured surrounding rock, conventional TSL materials require improved tensile–shear resistance, deformation compatibility, and support adaptability. Although fiber reinforcement is an effective method for enhancing polymer composites, systematic studies on the effects of fiber type and dosage in reactive polymer-based TSL materials remain limited. In this study, a commercially available two-component polyurea-silicate-based TSL matrix was reinforced with polyvinyl alcohol (PVA) fibers, polypropylene mesh fibers, and toughened polypropylene fibers at volume fractions of 0.25–1.50%. A stepwise experimental program, including uniaxial compression, variable-angle shear, tensile, circular-indenter buffered shear, and true triaxial tests, was conducted to evaluate the mechanical behavior and support-related performance of the fiber-reinforced TSL materials. The basic mechanical tests showed that the 0.75% toughened polypropylene fiber group maintained favorable compressive and shear resistance, achieving a cohesion of 8.65 MPa and an internal friction angle of 24.12°. PVA fibers exhibited higher tensile reinforcement efficiency at relatively low contents, with the 0.25% PVA fiber group reaching a peak tensile stress of 13.61 ± 1.00 MPa. The 1.0% PVA fiber group showed good deformation coordination, with a compressive strength of approximately 49.87 MPa. In the circular-indenter buffered shear test, the 1.0% PVA fiber group reached a peak load of 0.636 ± 0.055 kN and an absorbed energy of 3.118 ± 0.832 J at 10 mm displacement. Under true triaxial loading, the 1.0% PVA fiber group absorbed 311.4 J of energy at a displacement of 10 mm, approximately 5.5% higher than that of the 0.75% toughened polypropylene fiber group. Therefore, 1.0% PVA fiber reinforcement is recommended as the optimal reinforcement scheme for polymer-based TSL materials used in deep, fractured, and large-deformation coal mine roadways.

## 1. Introduction

Surface support for underground coal mine roadways commonly involves laying metal or fiber mesh [1], which is labor intensive and characterized by low automation and slow speed. TSL technology is a roadway surface support method in which a thin polymer-based layer is sprayed onto the surrounding rock surface. Owing to its rapid application, high degree of automation, and high equipment availability, TSL has gradually become a promising alternative to conventional metal mesh support in underground coal mines [2]. Furthermore, TSL technology is considered an active support system, meaning that after the sprayed material bonds with the surrounding rock to form a composite structure [3], it generates an active support effect through interaction with engineering applications, whereas metal mesh is generally regarded as a passive support system, whose primary function is to prevent the further collapse of loose surface rock.

Among the support mechanisms of TSL, the “load-bearing basket” effect is particularly critical for large-deformation and highly fractured roadways. When the surrounding rock deforms significantly or partially collapses, the TSL, together with the bonded rock blocks, forms a bulging composite structure that acts as a basket. Unlike loose debris, the rock fragments inside the basket are still subjected to compressive stresses from the surrounding rock, thereby maintaining a certain load-bearing capacity. In this mechanism, the tensile strength, shear resistance, and deformation compatibility of the TSL material play dominant roles.

Previous studies have investigated the support mechanisms, mechanical properties, and engineering applications of TSL [4,5,6]. Kanda and Stacey [7] reviewed its practical effectiveness in mining support and suggested that it can serve as a supplementary or alternative surface support system. Ozturk and Tannant [8] studied adhesive-strength testing methods for TSL and the influence of layer thickness on adhesion, indicating that interfacial bonding is a key factor in evaluating support performance. Yilmaz [9,10] conducted shear-bond and tensile strength tests on TSL products and reported that tensile strength, shear resistance, and interfacial bonding jointly determine their load-bearing capacity. Liang et al. [11] investigated the deformation behavior and application potential of polymer-based TSL in deep underground coal mines, demonstrating its adaptability to large-deformation roadways. Chen et al. [12] developed a polymer-modified TSL material for mine roadways and analyzed its basic properties. Dondapati et al. [13] examined the effect of fiber-reinforced TSL on the strength–deformability behavior of rock-like materials and coal, showing that fiber addition can improve post-peak deformation capacity. Kim et al. [14] and Kolapo et al. [15] further reviewed the development of TSL materials, testing methods, engineering applications, and remaining challenges, and pointed out that standardized testing methods, material–rock interaction, and long-term service performance still require further investigation. These studies have provided an important basis for understanding TSL support mechanisms and engineering applications. Systematic comparisons of different fiber types and dosages in reactive polymer-based TSL materials remain limited, especially regarding tensile–shear resistance, deformation compatibility, and energy-dissipation performance in deep large-deformation coal mine roadways.

The mechanical performance of TSL materials is generally improved through two approaches. The first approach is to develop polymer-based materials with higher strength, toughness, and deformation capacity. Recent studies on polymer-based TSL materials have shown that they can provide better early strength, tensile resistance, and deformation adaptability than cement-based TSL materials. Liang et al. [11] reported that after a shell-like support structure was formed by RPTSL, the yield strength and peak strength of the supported specimens increased by 20–45%, and the peak load of supported coal specimens increased by approximately 30%. These results indicate that polymer-based TSL materials can improve not only strength but also deformation control and energy absorption. Dondapati et al. [13] further showed that fiber-reinforced TSL can improve the strength–deformability behavior of rock-like materials and coal, especially in the post-peak stage. Existing studies have mainly focused on overall support performance or limited fiber-reinforced systems, while systematic comparisons of different fiber types and dosages in reactive polymer-based TSL materials remain insufficient. The second approach is fiber reinforcement, which can improve TSL performance at relatively low cost by bridging microcracks, delaying crack propagation, enhancing post-peak toughness, and improving energy absorption. In the present study, the optimized 1.0% PVA fiber-reinforced TSL material achieved a compressive strength of approximately 49.87 MPa, a peak tensile stress close to 14 MPa, and an energy absorption of 311.4 J at a displacement of 10 mm under true triaxial loading. Therefore, a systematic comparison of different fiber types and dosages is necessary to identify a suitable reinforcement scheme for polymer-based TSL materials used in deep large-deformation coal mine roadways.

Most existing studies on fiber-reinforced TSL have focused on cementitious or cement–polymer matrices, while the fiber reinforcement of reactive polymer-based TSL remains insufficiently investigated. The key scientific issue is not merely to screen fiber types and dosages, but to clarify how fiber characteristics and volume fractions affect the mechanical response, crack evolution, fracture morphology, and energy-absorption behavior of reactive polymer-based TSL under different loading conditions. To address this issue, three fiber types and six volume fractions were systematically compared through compression, variable-angle shear, and tensile tests to evaluate their effects on basic strength and deformation characteristics. Buffered shear tests and true triaxial tests were then conducted to further examine the coupled load-bearing behavior, failure process, and energy-dissipation capacity of the selected fiber-reinforced TSL materials under conditions closer to those of deep large-deformation roadways. This stepwise experimental framework was used to identify the optimal reinforcement scheme and clarify the reinforcement mechanism of fiber-reinforced reactive polymer-based TSL [14,15].

## 2. Experimental Study on the Basic Mechanical Properties of FRP

### 2.1. Materials and Methods for Basic Mechanical Tests

#### 2.1.1. Polymer Matrix and Fiber Materials

**Figure 1:**
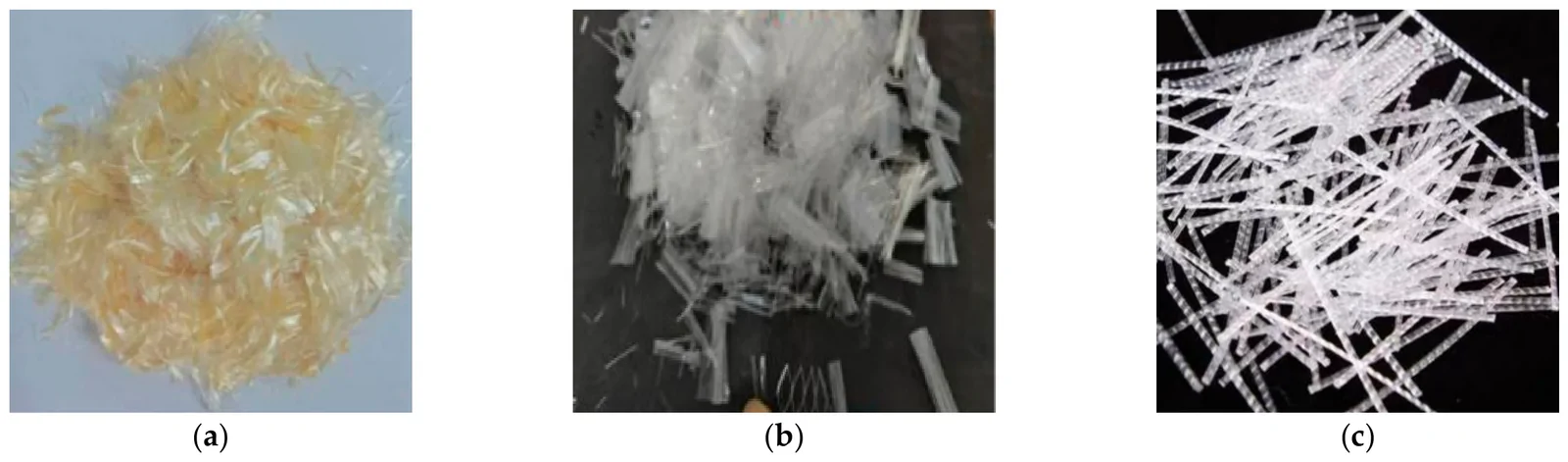
Fiber materials. (**a**) Polyvinyl alcohol fibers. (**b**) Polypropylene mesh fibers. (**c**) Toughened polypropylene fibers.

The matrix used in this study was MasterRoc MP 364 Flex, a commercially available two-component polyurea-silicate reactive polymer system marketed under the Master Builders Solutions brand. The system was supplied as MasterRoc MP 364 Flex Part A and MasterRoc MP 364 Flex Part B, hereafter referred to as Part A and Part B, respectively. This commercial matrix had previously been selected for rapid-curing TSL applications because its relatively low-exotherm reaction characteristics were consistent with the design objective of limiting curing-induced temperature rise [16]. In the present study, Parts A and B were used in their as-received condition, and no additional temperature-control additive, catalyst, or other chemical modifier was introduced into either component. Therefore, the modification investigated in this study refers exclusively to the incorporation of reinforcing fibers into the commercial A/B matrix. The densities of Parts A and B were 1500 and 1200 kg/m^3^, respectively, and their viscosities were 400–500 and 120–150 mPa·s, respectively. Parts A and B were mixed at a volume ratio of 1:1. The initial curing time after mixing was 90 ± 30 s, the specimens developed sufficient early strength for demolding within approximately 2 min, and all specimens were subsequently cured under the same ambient laboratory conditions for 7 days before testing or further machining. The complete molecular formulation and exact proportions of proprietary constituents were not available in the archived product documentation and were not independently determined in this study. Nevertheless, the commercial product name, brand, component designations, basic physical properties, mixing ratio, specimen-preparation procedure, and curing conditions are reported to enable reproduction using the same commercial matrix system. The selection of fiber materials primarily considered availability and widespread applicability. Three reinforcing materials widely used in polymer and cementitious composites were selected: polyvinyl alcohol (PVA) fibers, polypropylene mesh fibers, and toughened polypropylene fibers, as shown in Figure 1.

Recent studies on PVA-based polymer systems and fiber-reinforced polymer composites have also shown that polymer fibers can improve toughness, interfacial interaction, and deformation capacity, providing further support for selecting PVA and polypropylene-based fibers as reinforcement materials [17,18,19,20].

The three fibers used in this study were commercially available industrial fibers selected according to their availability and common use in polymer and cementitious composite reinforcement. The parameters listed in Table 1 are nominal values obtained from publicly available supplier product specifications and technical information. Because the fibers were purchased through commercial distributors and complete batch certificates were not available, the specific manufacturer, batch number, density, fiber length, and detailed surface-treatment information could not be verified from the archived procurement records and are therefore not reported. No additional surface treatment was conducted before mixing, and all fibers were used in the as-received state. To avoid overinterpretation of the fiber geometry, the diameter values in Table 1 should be regarded as nominal or equivalent diameters. Although some supplier-related information is limited, all specimens were prepared using fibers from the same procurement batch for each fiber type, ensuring the comparability of the experimental results. Their main parameters are listed in Table 1.

As shown in Table 1, the three types of fibers exhibit significant differences in terms of elastic modulus, elongation at break, diameter, and tensile strength, providing a foundation for subsequent analysis of the reinforcing effects of fibers with different characteristics.

In the engineering applications considered in this study, 5–8 mm was taken as the target thickness range of the polymer-based TSL layer. This thickness range was used to define the thin-layer spraying condition under which the material should satisfy the requirements of workability, flowability, formability, and fiber dispersion. Under such thin-layer spraying conditions, excessive fiber addition may reduce the flowability and formability of the mixture, whereas too low a fiber content may be insufficient to form an effective reinforcement network. Accordingly, the fiber loading was set at six volume fractions: 0.25%, 0.5%, 0.75%, 1.0%, 1.25%, and 1.5%.

The fiber loading range was determined based on preliminary mixing and dispersion trials, in which workability, flowability, visible dispersion quality, and specimen formability were considered. The acceptability of the mixture was judged according to whether the fibers could be incorporated into the A/B polymer matrix within the available mixing time before rapid curing, whether obvious fiber bundles, floating fibers, or local agglomerates were observed after mixing, whether the mixture could be poured into the molds without severe viscosity increase or loss of formability, and whether the demolded specimens showed macroscopic defects such as large pores, exposed fiber clusters, or nonuniform fiber distribution. When the PVA fiber content exceeded 1.5%, visible fiber agglomeration and reduced flowability were observed because of the low density and large number of fine filaments. For the polypropylene mesh fibers and toughened polypropylene fibers, contents lower than 0.25% resulted in sparse and discontinuous fiber distribution, which was considered insufficient for forming an effective reinforcement network. Therefore, the content range of 0.25–1.5% was selected for the subsequent uniaxial compression, variable-angle shear, tensile, buffered shear, and true triaxial tests. It should be noted that the preliminary screening was based on macroscopic workability and dispersion observations rather than quantitative microscopic image analysis; quantitative characterization of fiber dispersion, agglomeration ratio, and pore structure will be further investigated in future work.

The commercial MasterRoc MP 364 Flex A/B matrix described in Section 2.1.1 exhibited rapid early-strength development. After Parts A and B were mixed at a 1:1 volume ratio, the material reacted mildly and developed sufficient early strength for demolding within approximately 2 min. Demolding time does not represent the final curing age used for mechanical testing. After demolding and trimming, all specimens were cured under the same ambient laboratory conditions for 7 days before testing or further machining. The same curing age, preparation procedure, and testing schedule were adopted for all fiber types and contents to ensure comparability among different groups. Environmental temperature and humidity were not taken as independent variables in this study; instead, specimen preparation, demolding, trimming, curing, and machining were conducted following the same fixed procedure. Therefore, the comparative results mainly reflect the effects of fiber type and fiber content on the mechanical behavior of the reactive polymer-based TSL materials [16].

#### 2.1.2. Uniaxial Compression Test

**Figure 2:**
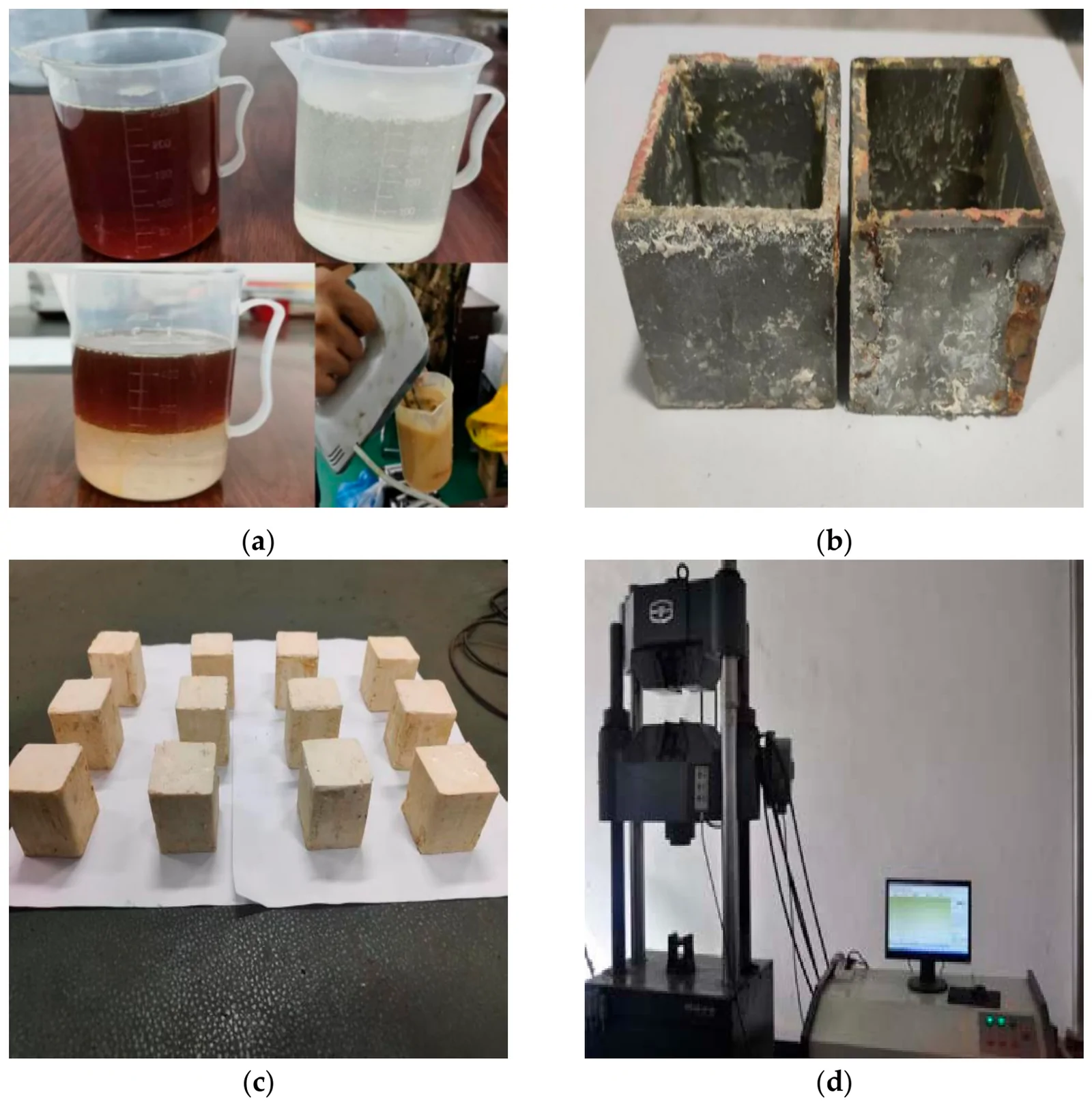
Specimen preparation and testing. (**a**) Measurement and mixing of two-component polyurethane material. (**b**) Mold for compressive test specimens. (**c**) Some of the compressive test specimens. (**d**) WAW-600C electro-hydraulic servo universal testing machine.

Test specimens were prepared in accordance with ASTM D695 [21] for compression testing of polymer-based TSL materials. Components A and B were mixed at a 1:1 volume ratio, and fibers were added at volume fractions of 0.25%, 0.5%, 0.75%, 1.0%, 1.25%, and 1.5% relative to the total mixture volume, as shown in Figure 2a. For each fiber type and content, three parallel specimens were prepared. To ensure traceability, all specimens were coded according to the format Fiber–Content–Test–Replicate, where Fiber represents the fiber type, Content represents the fiber volume fraction, Test represents the test type (C = compression), and Replicate represents the specimen number. For example, PVA-1.00-C-01 denotes the first compression specimen containing 1.00% PVA fiber, TPP-0.75-C-02 denotes the second compression specimen containing 0.75% toughened polypropylene fiber, and M-0-C-01 denotes the first unmodified matrix specimen. Load and displacement data were recorded by the WAW-600C testing system using the same acquisition setting for all specimens. Compressive stress was calculated as the axial load divided by the original cross-sectional area, and axial strain was calculated as the axial displacement divided by the original specimen height. Data were considered valid only when the specimen had no obvious preparation defects, the loading process and data record were complete, and failure was not caused by abnormal end contact, eccentric loading, or equipment interruption.

For each batch, MasterRoc MP 364 Flex (BASF SE, Ludwigshafen, Germany) Part A and Part B were measured at a 1:1 volume ratio and mixed using a mechanical stirrer. According to the reported technical data and previous characterization of the same A/B system [16], the viscosity of Part A was 400–500 mPa·s, the viscosity of Part B was 120–150 mPa·s, and the initial curing time after mixing was 90 ± 30 s. During mixing, the stirring speed was maintained at 500 ± 50 rpm, corresponding to an actual rotational speed range of 400–500 rpm [22]. This speed range was determined based on preliminary mixing trials, as it provided sufficient shear force to promote fiber dispersion within the matrix while minimizing excessive air entrainment, fiber entanglement, and premature local agglomeration during the short working time before rapid curing. The fibers were added in three separate portions at the specified volume fractions of 0.25–1.5%, and stirring continued for approximately 30 s to promote uniform dispersion before curing. A propeller-type stirrer with a diameter of 40 mm was used to generate a stable vortex and further reduce fiber entanglement. It should be noted that the viscosity of each fiber-reinforced mixture was not independently measured using a rheometer in this study. Therefore, workability- and sprayability-related suitability were evaluated under fixed mixing conditions according to whether the mixture could be uniformly stirred within the available working time, whether severe viscosity increase or poor mold filling occurred, and whether obvious fiber bundles, floating fibers, local agglomerates, or visible specimen defects were observed.

Specimens were cast using 40 mm cubic iron molds, as shown in Figure 2b. Lubricating oil was applied to the mold contact surfaces to facilitate demolding. After casting, the specimens could be demolded within approximately 2 min due to the rapid early-strength development of the A/B polymer system. After demolding, excess material was trimmed, and the loading surfaces were finished to ensure good contact with the loading platens. Specimens with obvious edge damage, uneven loading surfaces, pores, or visible molding defects were excluded. All specimens were cured under the same ambient laboratory conditions for 7 days before testing, and all groups were tested at the same curing age. The compression tests were performed using a WAW-600C microcomputer-controlled electro-hydraulic servo universal testing machine (Jinan Shijin Group Co., Ltd., Jinan, China), as shown in Figure 2d. A displacement-controlled loading mode with a loading rate of 1 mm/min was adopted to ensure stable and continuous loading throughout the test process, while minimizing dynamic effects and allowing accurate capture of the complete stress–strain response and failure behavior of the specimens.

#### 2.1.3. Variable-Angle Shear Test

**Figure 3:**
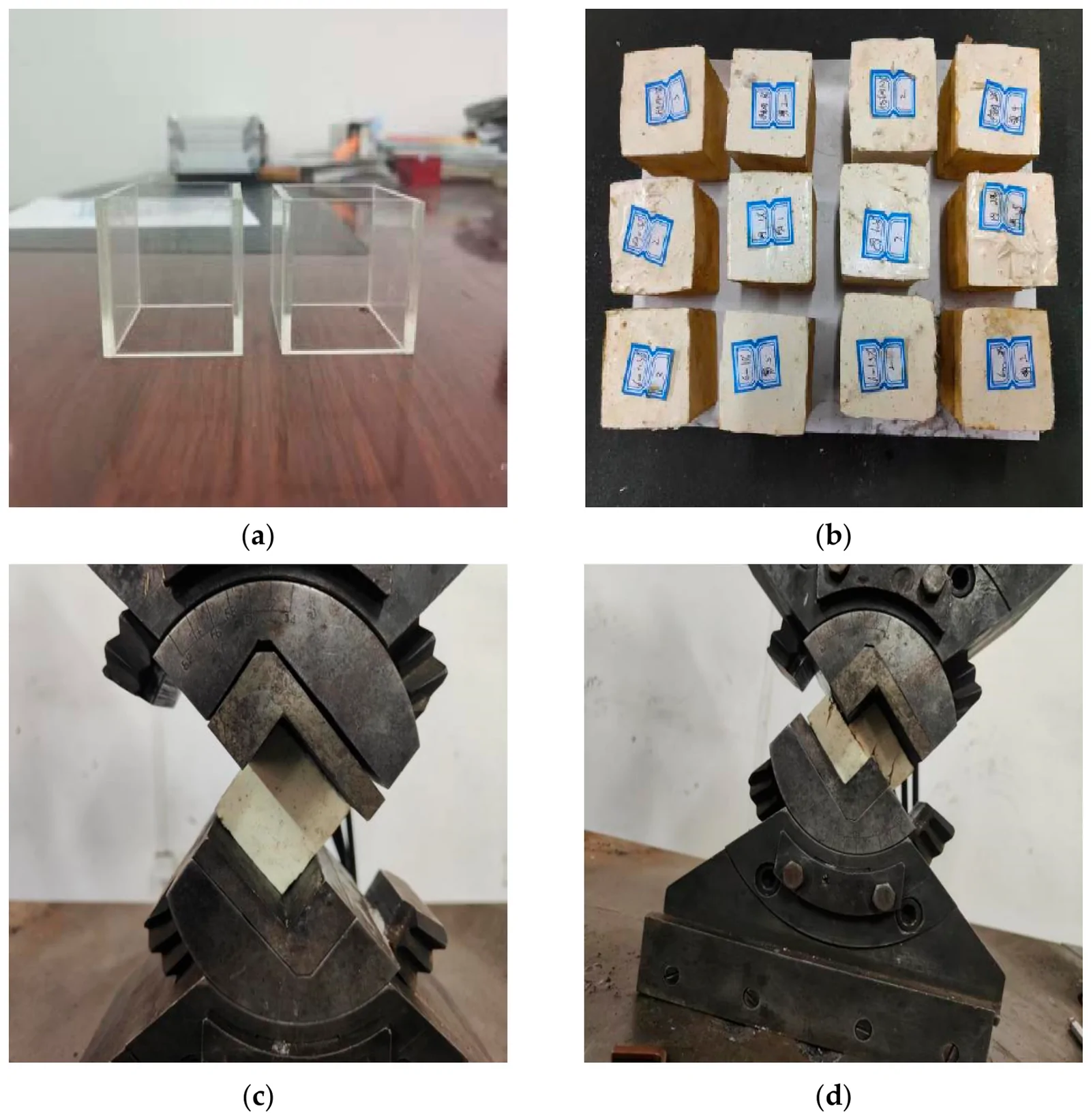
Preparation and testing of shear specimens. (**a**) Cubic acrylic mold (50 mm). (**b**) Some of the shear specimens. (**c**) Variable-angle shear fixture. (**d**) Shear test.

In accordance with GB/T 23561.11-2010 [23], Methods for Determining the Physical and Mechanical Properties of Coal and Rock—Part 11: Method for Determining Shear Strength of Coal and Rock, the variable-angle shear specimens were cast using 50 mm cubic acrylic molds, as shown in Figure 3a. The specimen preparation and curing procedures were the same as those used for the compression specimens, and the prepared shear specimens are shown in Figure 3b. For each fiber type, fiber content, and shear angle, three parallel specimens were prepared and tested. The test was conducted using a WAW-600C microcomputer-controlled electro-hydraulic servo universal testing machine and variable-angle shear grips, with shear angles of 42°, 50°, 58°, 66°, and 74° [24]. A displacement-controlled loading mode was adopted at a loading rate of 1 mm/min, and the testing process is shown in Figure 3c,d. For each specimen, the peak shear failure load $P_{s}$ was recorded. The normal stress $\sigma_{s}$ and shear strength $\tau_{s}$ on the shear plane were calculated as follows:

(1) $$\sigma_{s}=\frac{P_{s}}{F_{s}}\cdot \cos\left(\alpha_{s}\right)\times 10$$

(2) $$\tau_{s}=\frac{P_{s}}{F_{s}}\cdot \sin\left(\alpha_{s}\right)\times 10$$

where $\sigma_{s}$ is the normal stress on the shear plane (MPa), $\tau_{s}$ is the shear strength on the shear plane (MPa), $P_{s\;}$is the shear failure load (kN), $F_{s}$ is the shear-plane area (cm^2^), and $\alpha_{s}$ is the angle between the shear specimen and the horizontal plane. The cohesion (c) and internal friction angle φ were then obtained by fitting the Mohr–Coulomb relationship:

(3) $$\tau_{s}=c+\sigma_{s}tan\left(\varphi \right)\;$$

Although GB/T 23561.11-2010 is originally intended for coal and rock materials, it was adopted in this study because no specific standard is currently available for variable-angle shear testing of polymer-based TSL materials. The purpose of using this standard was to provide a controlled and comparable shear-loading method under the same specimen geometry, shear angles, and loading conditions.

#### 2.1.4. Tensile Test

Tensile tests were conducted with reference to the loading and data-processing principles of ASTM D638 [25]. It should be noted that the specimens used in this study were not the standard flat dog-bone specimens specified in ASTM D638, but customized cylindrical reduced-section tensile specimens designed for the rapid-curing polymer-based TSL material. Each specimen had a total length of 200 mm, with two gripping ends of 30 mm in diameter and 50 mm in length, and a reduced test section of 20 mm in diameter and 100 mm in length. This geometry was adopted to facilitate casting and demolding, improve gripping stability, and promote tensile failure within the reduced test section. Therefore, the tensile results are mainly used for comparative evaluation of different fiber types and contents under the same specimen geometry and loading conditions. The results should not be directly regarded as standard ASTM D638 tensile properties.

**Figure 4:**
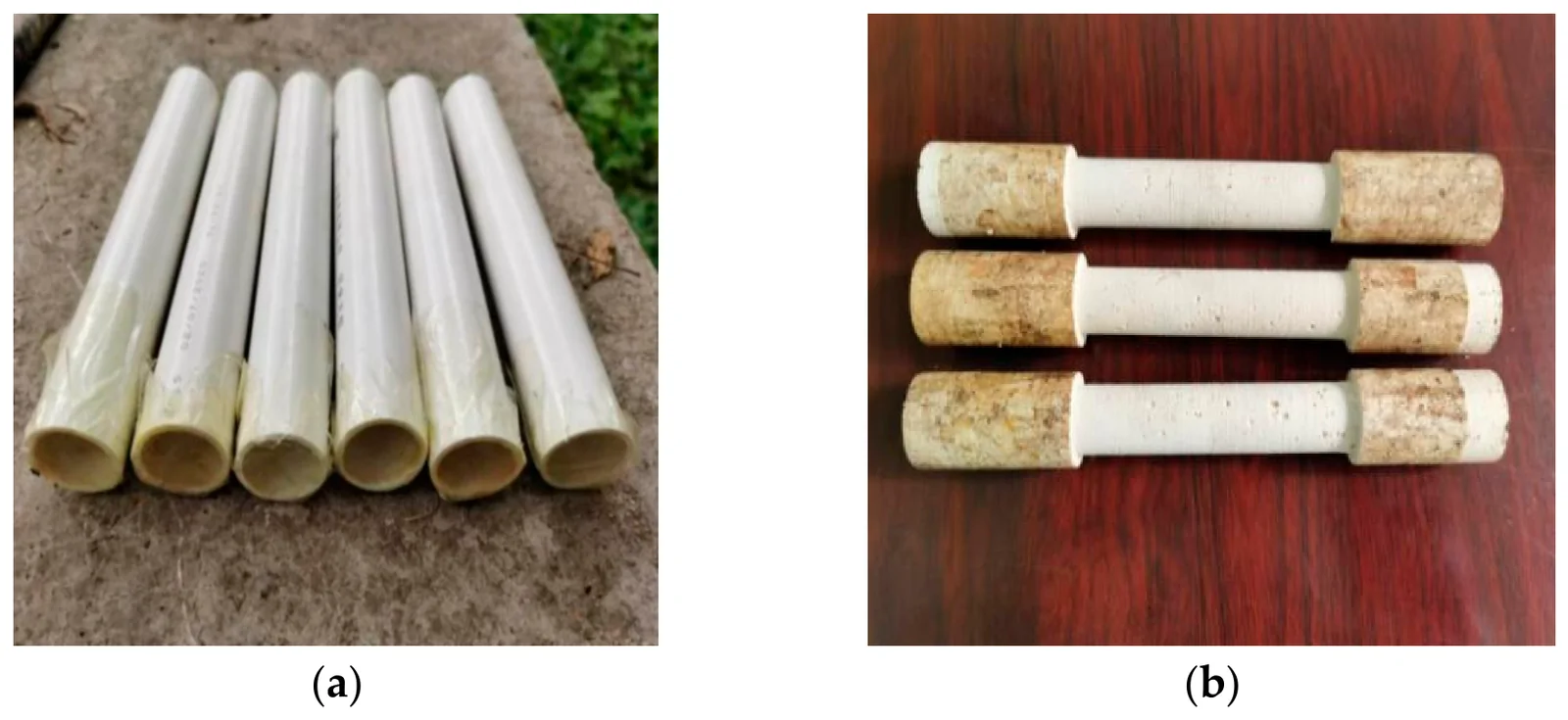
Preparation and fabrication of tensile specimens. (**a**) PVC tube tensile mold. (**b**) Prepared tensile test specimen.

The specimen preparation mold consisted of a PVC tube with a diameter of 30 mm and a length of 200 mm. Lubricating oil was applied to the inner surface of the PVC tube, and one end of the tube was sealed with adhesive tape, as shown in Figure 4. The mixed polymer material was then poured into the PVC tensile mold. After demolding, the specimens were cured under the same ambient laboratory conditions for 7 days. They were then machined into the required cylindrical reduced-section tensile specimens using a milling machine, as shown in Figure 4a,b.

**Figure 5:**
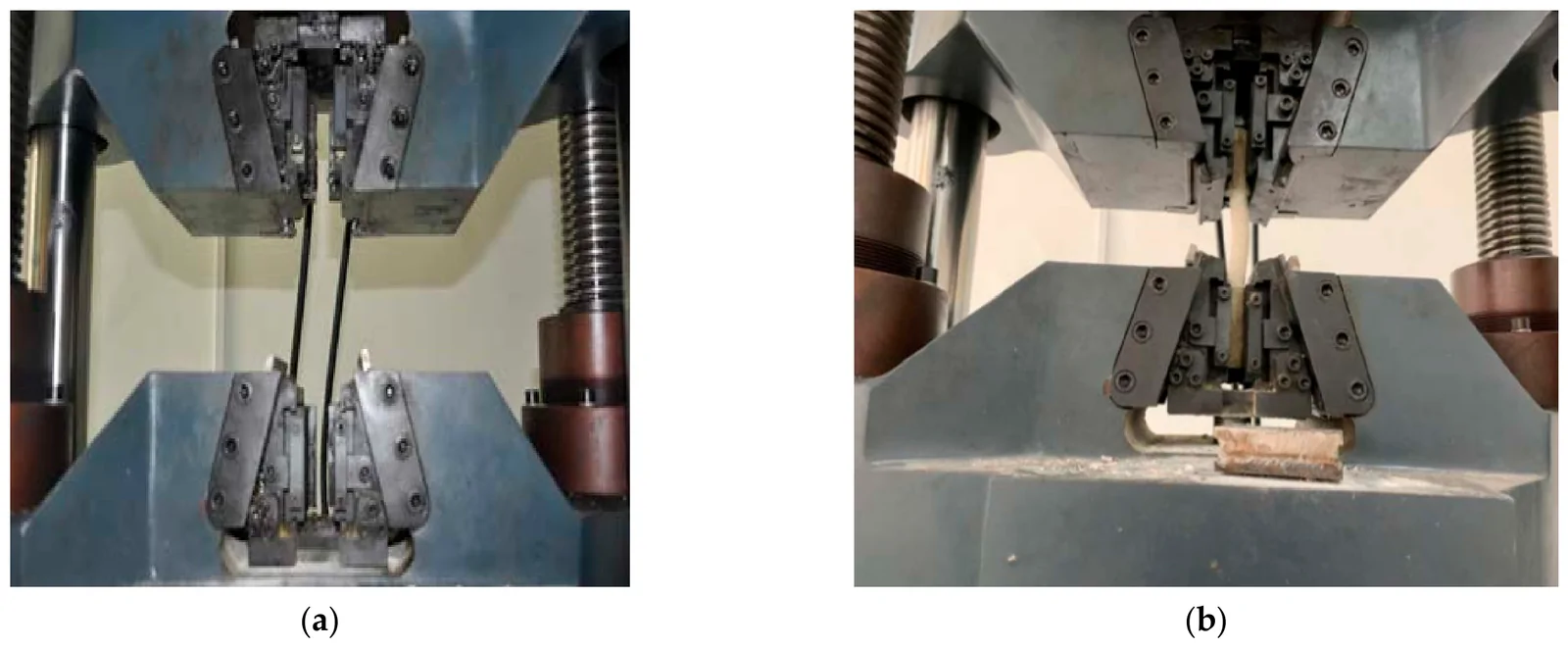
Tensile test. (**a**) Tensile testing fixture. (**b**) Tensile testing process.

The WAW-600C microcomputer-controlled electro-hydraulic servo universal testing machine was again selected as the experimental testing machine, and tensile grips were used to clamp the ends of the tensile specimens. The loading mode was displacement control, with a loading rate of 1 mm/min. The tensile testing process is shown in Figure 5a,b.

The engineering tensile stress and engineering tensile strain were calculated as follows:

(4) $$\sigma_{t}=\frac{P_{t}}{A_{t}}$$

(5) $$A_{t}=\frac{\pi d^{2}}{4}$$

(6) $$\epsilon_{t}=\frac{\Delta L}{L_{0}}$$

where $\sigma_{t}$ is the engineering tensile stress, $P_{t}$ is the tensile load, $A_{t}$ is the original cross-sectional area of the reduced test section, d is the diameter of the reduced test section, $\epsilon_{t}$ is the engineering tensile strain, ΔL is the recorded tensile displacement used for strain calculation, and $L_{0}$ is the original length of the reduced test section. In this study, d = 20 mm and $L_{0}$ = 100 mm were used for the reduced test section. The tensile toughness was determined as the area under the tensile stress–strain curve up to the final recorded fracture/end strain and was calculated from the discrete test data using the trapezoidal rule. For plotting and tabulation, tensile strain was expressed as a percentage, whereas the strain used for toughness calculation was expressed in decimal form. Therefore, the tensile toughness has the unit of MPa, equivalent to MJ/m^3^.

The tensile test, together with the uniaxial compression and variable-angle shear tests, completed the basic mechanical screening of the modified TSL materials under single loading conditions. The selected candidate schemes were then further evaluated by buffered shear and true triaxial compression tests to assess their coupled load-bearing, energy-dissipation, and stress-environment adaptability [11,14,26]. All tests were conducted under low-rate displacement-controlled loading, and the possible rate-dependent behavior of polymer-based TSL materials should be further investigated in future work.

### 2.2. Results and Analysis

#### 2.2.1. FRP Uniaxial Compression Test

For each fiber type and content, three parallel compression specimens were tested. The stress–strain curves presented in this section represent the arithmetic mean of the valid parallel specimens for each group. For clarity, the abbreviations used in the curve labels are defined as follows: PVA denotes polyvinyl alcohol fiber, PPMF denotes polypropylene mesh fiber, TPPF denotes toughened polypropylene fiber, M denotes the unreinforced matrix material, and C denotes the compression test. To avoid overcrowding in the figures, replicate numbers were omitted from the curve labels. For example, PVA-C-0.25% denotes the average compression response of three valid PVA-fiber-reinforced specimens with a fiber volume fraction of 0.25%, whereas M-C-0% denotes the average compression response of the unreinforced matrix specimens.

**Figure 6:**
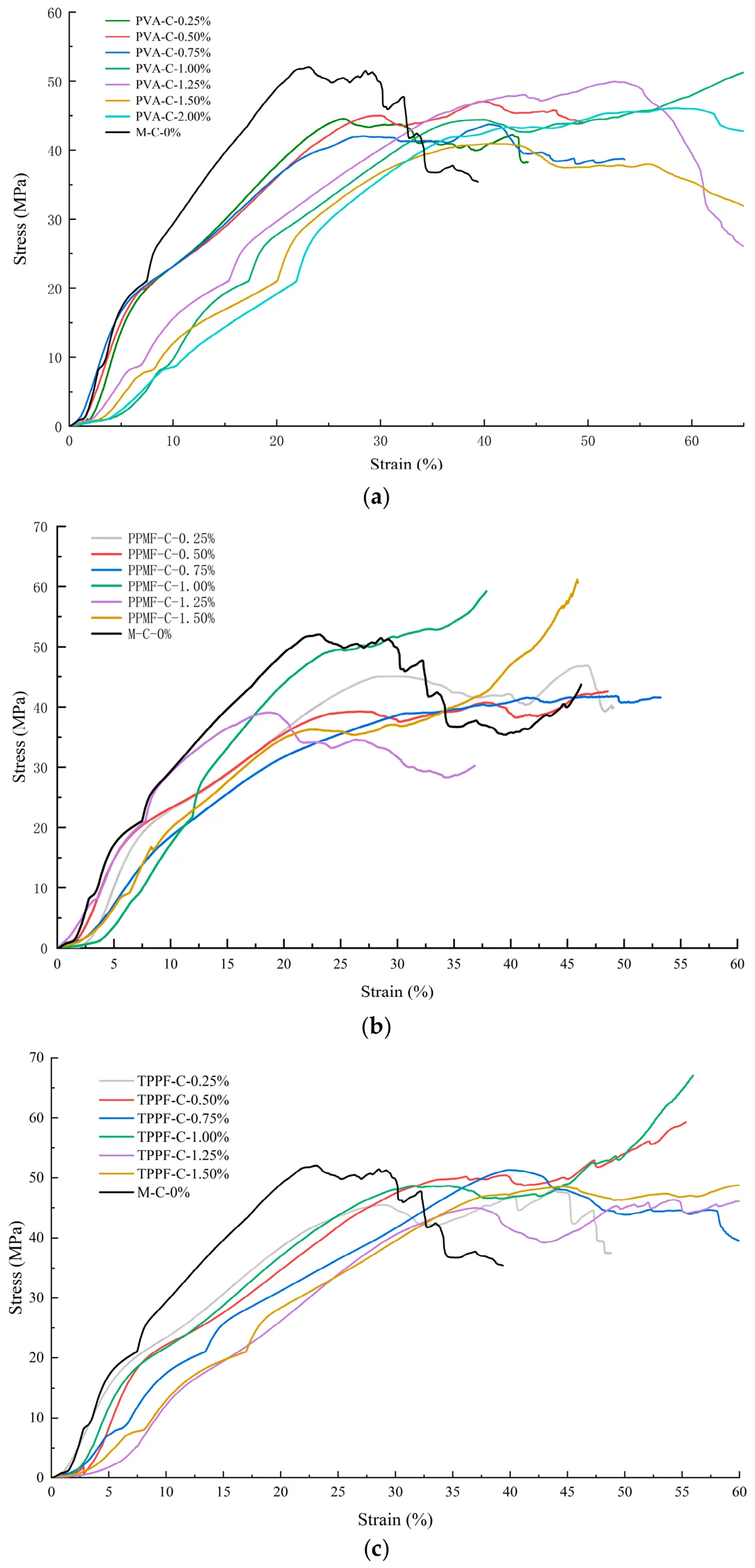
Stress–strain curves of three fibers at different contents. (**a**) Compressive stress–strain curves of PVA-reinforced TSL. (**b**) Compressive stress–strain curves of PPMF-reinforced TSL. (**c**) Compressive stress–strain curves of TPPF-reinforced TSL.

**Figure 7:**
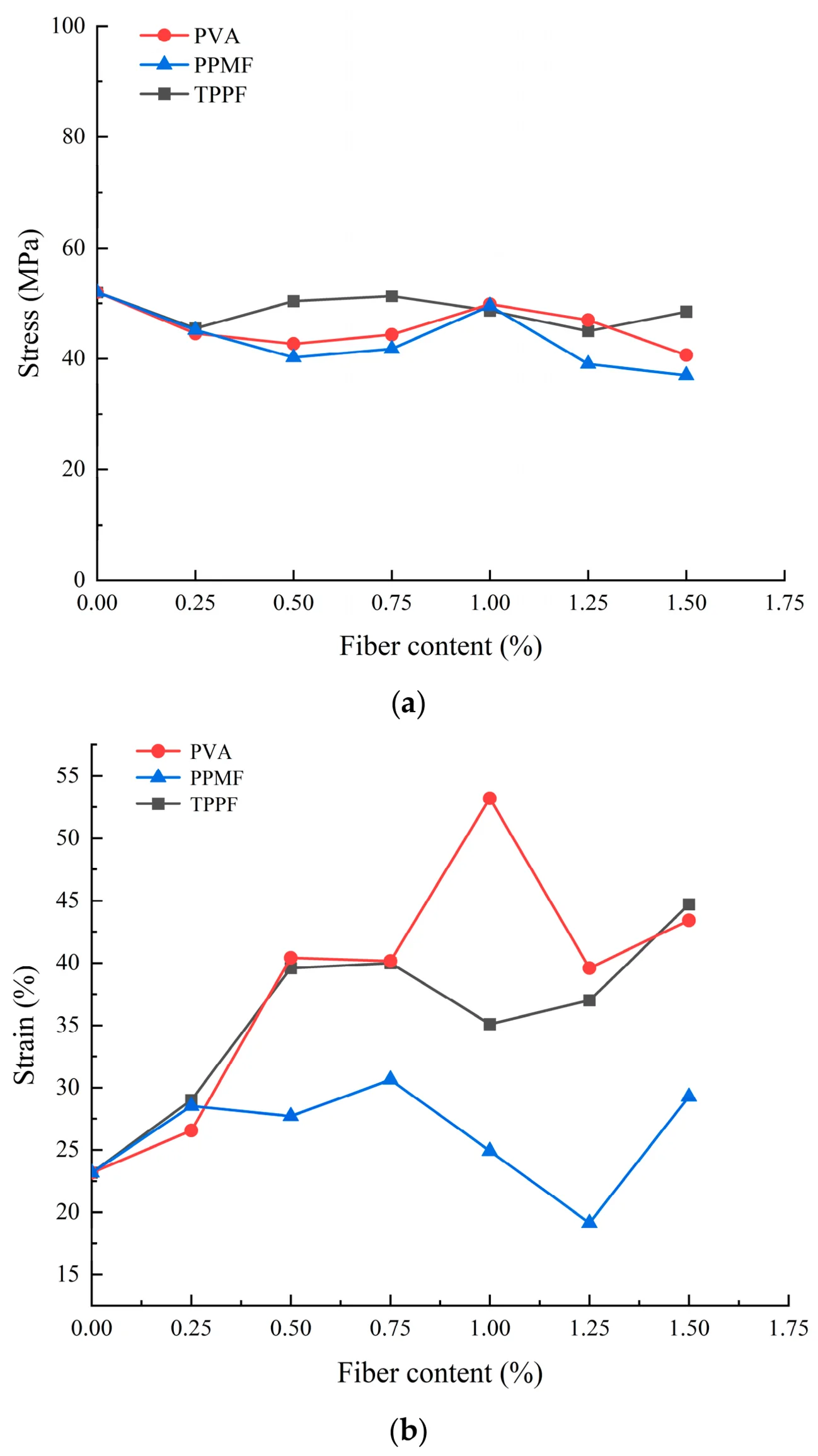
Stress–strain-content plots for different types of fibers. (**a**) Peak stress versus fiber content for different fibers. (**b**) Plot of peak strain versus fiber content for different fibers.

As shown in Figure 6, the peak stress of the raw material specimen is approximately 52 MPa. After fiber incorporation, the compression failure process of each specimen group still undergoes the stages of initial compaction, near-linear elasticity, damage propagation, and post-peak failure; however, the peak strain increases, and the post-peak stress decay slows down. This indicates that the primary effect of the fibers on the material is not to significantly increase the peak compressive strength, but rather to improve its post-peak stability and deformation coordination capability. The variation in peak stress and peak strain in specimens with different fibers as a function of fiber content is shown in Figure 7 and Table 2.

To further clarify the repeatability of the compression test results, the peak compressive stress values of three parallel specimens for each group were extracted from the corresponding stress–strain curves. The results are summarized as mean ± standard deviation, as shown in Table 2.

As shown in Figure 7 and Table 2, fiber modification had a more pronounced effect on peak strain than on peak compressive stress. The peak compressive stress of the unreinforced matrix was 52.00 MPa. After fiber incorporation, most fiber-reinforced groups exhibited peak compressive stresses comparable to that of the matrix, with slight variations depending on fiber type and content. Among the PVA-reinforced groups, PVA-C-1.00% exhibited the highest peak compressive stress of 49.87 MPa. For the PPMF-reinforced groups, PPMF-C-1.00% reached 49.54 MPa. Among the TPPF-reinforced groups, TPPF-C-0.75% exhibited the highest peak compressive stress of 51.30 MPa, which was very close to that of the matrix.

The peak strain showed a more evident improvement after fiber addition. The peak strain of the unreinforced matrix was 23.17%, whereas PVA-C-1.00% reached the maximum peak strain of 53.19%, approximately 2.30 times that of the matrix. The maximum peak strain of the PPMF-reinforced groups was 30.65% at PPMF-C-0.75%. The TPPF-C-0.75% group reached a peak strain of 40.00%, corresponding to an increase of approximately 72.6% compared with the matrix. These results indicate that fiber reinforcement mainly enhanced the pre-failure deformation capacity and post-peak stability of the polymer-based TSL material. Considering both peak stress and peak strain, PVA-C-1.00% showed the greatest improvement in deformation capacity, while TPPF-C-0.75% maintained the highest compressive strength retention.

The above results indicate that fiber modification did not significantly increase the peak compressive strength of the polymer-based TSL material. Its main effect was to improve the pre-failure deformation capacity and post-peak stability. This improvement is related to the bridging and restraining effects of fibers during crack development. Excessive fiber content may reduce the uniformity of fiber distribution and introduce local defects, resulting in a decrease in compressive performance.

**Figure 8:**
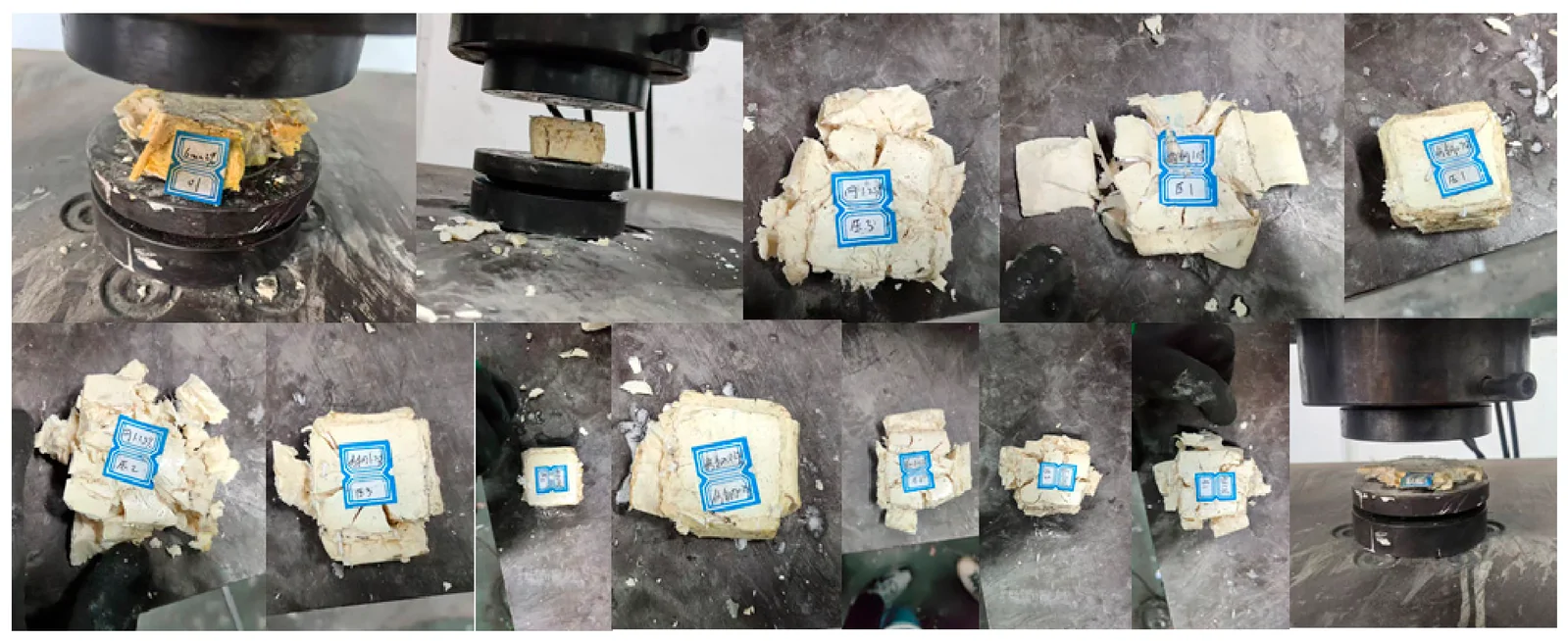
Failure modes of compressive specimens.

Figure 8 shows the macroscopic post-failure morphology of the specimens after compression. The unreinforced matrix specimens showed localized fragmentation, corner spalling, and rapid crack development after reaching the peak load, indicating a relatively sudden loss of integrity at the macroscopic scale. After fiber incorporation, the failed specimens generally retained better overall integrity, and the cracks appeared more distributed rather than being concentrated in a single localized failure zone. These macroscopic observations suggest that the fibers may have contributed to crack bridging and deformation restraint during the compression failure process. However, it should be noted that the present interpretation is mainly based on macroscopic post-failure morphology. No SEM or microscopic fracture-surface observation was conducted in this study because of experimental-condition limitations. Therefore, the detailed microscopic mechanisms of fiber–matrix bonding, crack bridging, and energy dissipation require further investigation in future work.

Different fiber types exert varying regulatory effects on the failure process. Due to their high strength, fine diameter, and relatively good dispersion, polyvinyl alcohol fibers exhibit a significant inhibitory effect on microcrack initiation and propagation, and thus perform better in improving the specimens’ deformation capacity and post-failure integrity. Toughened polypropylene fibers, on the other hand, offer certain advantages in enhancing peak load-bearing capacity and improving post-peak stability; their mechanism of action is primarily reflected in deformation coordination and enhanced energy dissipation during the late stages of crack propagation. Since polypropylene mesh fibers struggle to form a uniform and continuous reinforcement structure within the matrix, their inhibitory effect on crack propagation is relatively limited; consequently, their overall modification effect is inferior to that of the former two types.

In summary, the macroscopic failure morphology suggests that appropriate fiber types and contents may improve the post-failure integrity of polymer-based TSL materials and reduce the degree of localized fragmentation after compression failure. Because this interpretation is based mainly on macroscopic observations, it should be regarded as a phenomenological indication rather than direct microscopic evidence of a change in fracture mechanism. Based on the combined peak stress and peak strain results, PVA-C-1.00% showed the largest increase in peak strain, reaching 53.19%, which was approximately 2.30 times that of the unreinforced matrix. TPPF-C-0.75% maintained the highest peak compressive stress among the fiber-reinforced groups, reaching 51.30 MPa, while its peak strain increased to 40.00%. In comparison, the maximum peak strain of the PPMF-reinforced groups was 30.65%, and their peak compressive stress was generally lower than that of the unreinforced matrix. Therefore, PVA-C-1.00% and TPPF-C-0.75% were selected as the candidate fiber-modification schemes for subsequent tests.

#### 2.2.2. Analysis of Variable-Angle Shear Test Results

**Figure 9:**
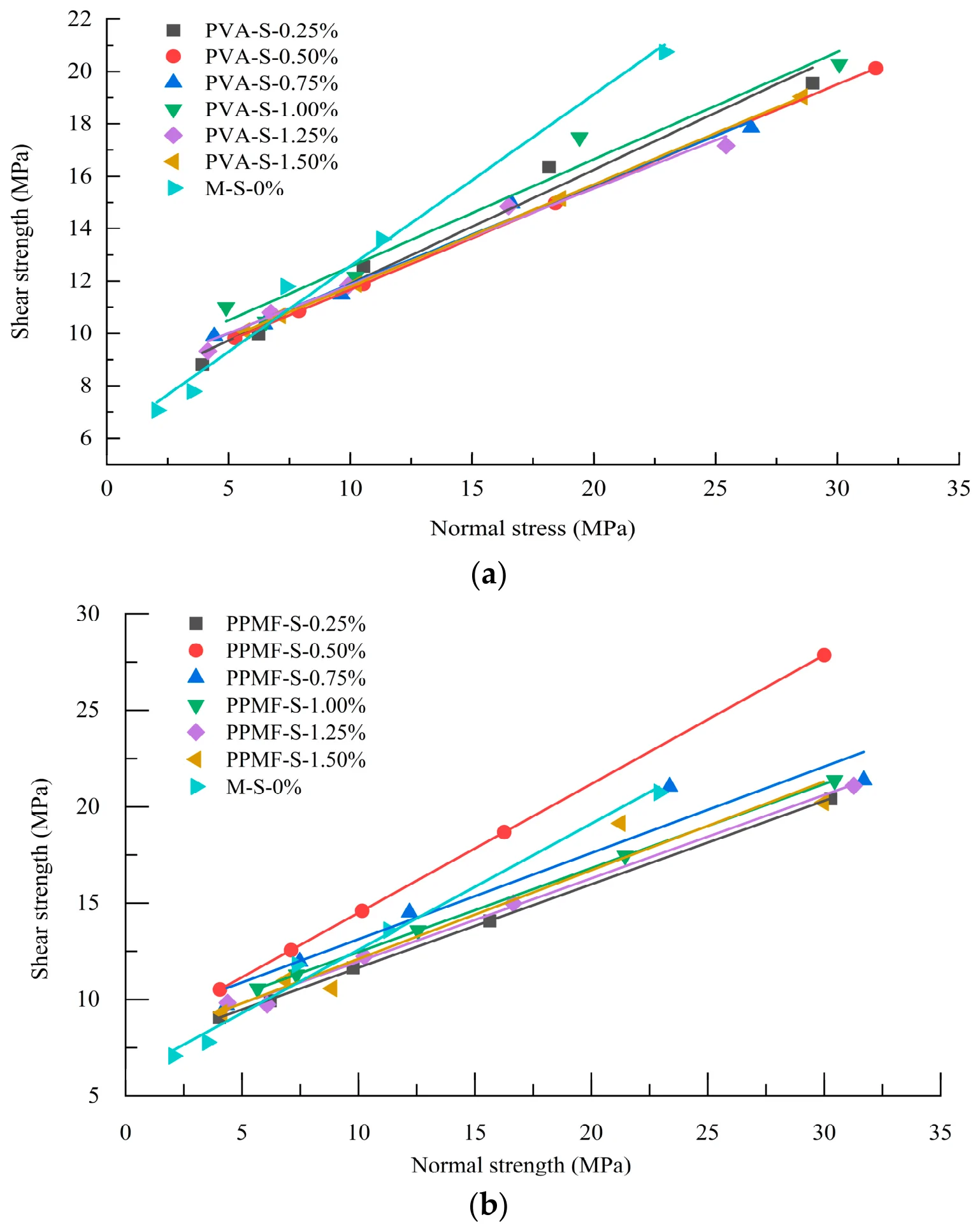

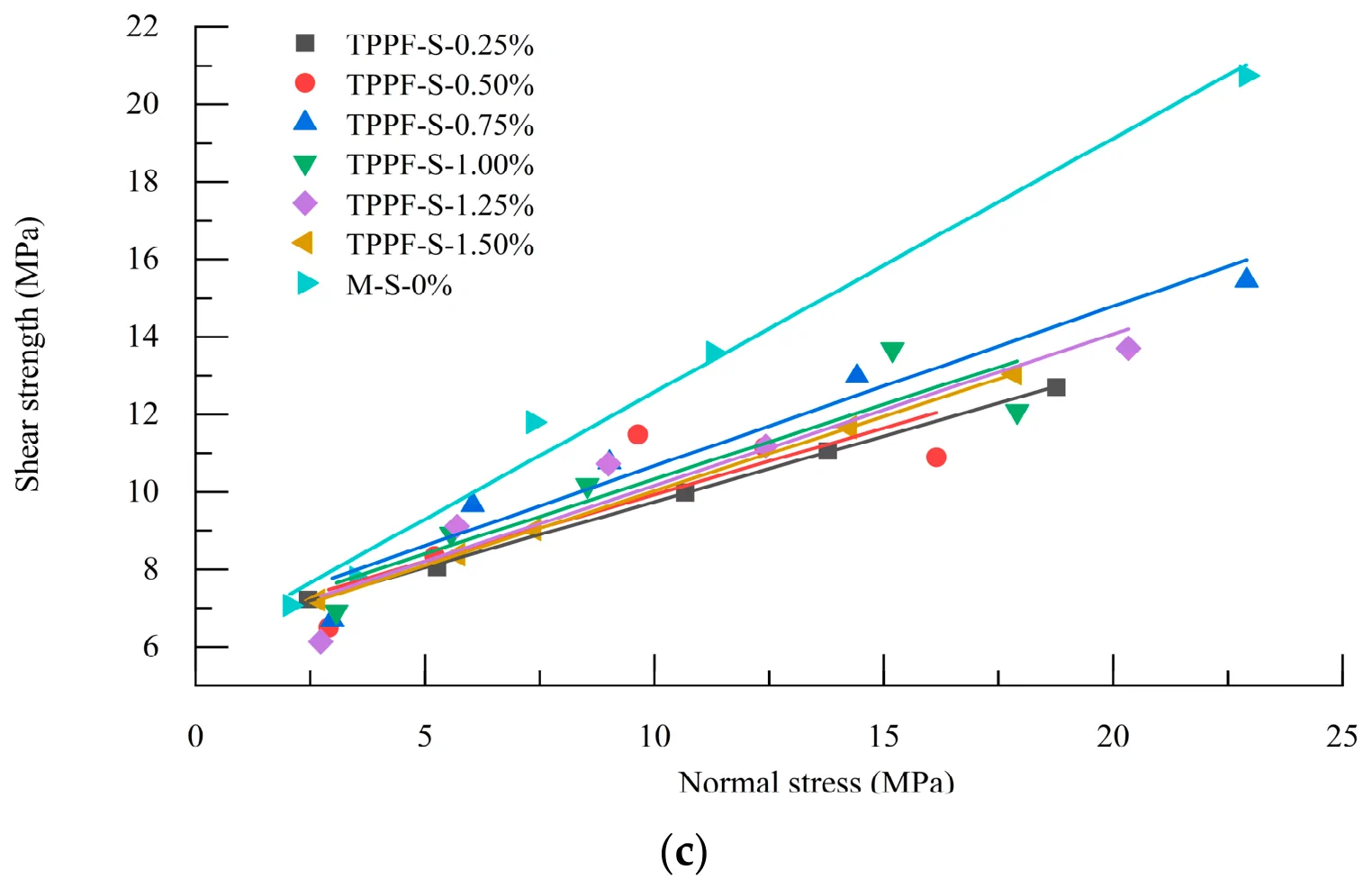
Shear strength curves of specimens with different fibers. (**a**) Shear curve of polyvinyl alcohol fiber. (**b**) Shear curve of polypropylene mesh fiber. (**c**) Shear curve of toughened polypropylene fiber.

For each fiber type, fiber content, and shear angle, three parallel specimens were tested. To ensure consistency among the text, Table 3 and Table 4, and Figure 9, the material groups were uniformly coded as Fiber–S–Content, where Fiber denotes the fiber type, S denotes the variable-angle shear test, and Content denotes the fiber volume fraction. In this coding system, PVA, PPMF, and TPPF represent polyvinyl alcohol fiber, polypropylene mesh fiber, and toughened polypropylene fiber, respectively, while M represents the unmodified matrix material. For example, PVA-S-1.00% represents the PVA-fiber-reinforced polymer-based TSL material with a fiber volume fraction of 1.00% under variable-angle shear testing, whereas M-S-0% represents the unmodified matrix material.

The normal stress and shear strength at five shear angles were calculated according to Equations (1) and (2), respectively, following the variable-angle shear testing method commonly adopted for evaluating the shear mechanical behavior of thin spray-on liner materials and cementitious composites [24,27]. The calculated results are summarized in Table 3 as mean ± standard deviation (*n* = 3). Based on the mean values of normal stress and shear strength, the Mohr–Coulomb fitting curves were established to characterize the shear failure behavior of the materials under different normal stress conditions, as shown in Figure 9 [28]. The cohesion and internal friction angle were subsequently determined by fitting the Mohr–Coulomb relationship shown in Equation (3), and the corresponding fitting parameters are listed in Table 3. The Mohr–Coulomb criterion has been widely used to evaluate the shear resistance and interfacial mechanical properties of rock-support materials and polymer-based reinforcement systems [29,30].

As shown in Figure 9 and Table 4, all groups exhibited approximately linear relationships between shear strength and normal stress, indicating that the shear behavior of the fiber-reinforced polymer-based TSL materials can be reasonably described by the Mohr–Coulomb criterion, which has been widely applied to characterize the shear failure behavior of cementitious and polymer-based composite materials under combined normal and shear loading conditions [24,27]. For the PVA-reinforced groups, the cohesion ranged from 7.55 to 8.45 MPa, and PVA-S-1.00% showed the highest cohesion, indicating a stronger contribution to shear resistance. This result is consistent with previous studies reporting that PVA fibers can effectively improve interfacial bonding and enhance crack-bridging capacity, thereby increasing the shear resistance of composite materials [28].

For the PPMF-reinforced groups, the cohesion values were generally lower than those of the PVA- and TPPF-reinforced groups, suggesting that polypropylene mesh fibers were less effective in improving the shear resistance of the polymer-based TSL material. This may be attributed to the relatively weaker interfacial adhesion between polypropylene fibers and the surrounding matrix, which limits stress transfer efficiency during shear deformation, as reported in previous investigations on polypropylene-reinforced cementitious composites [29].

**Figure 10:**
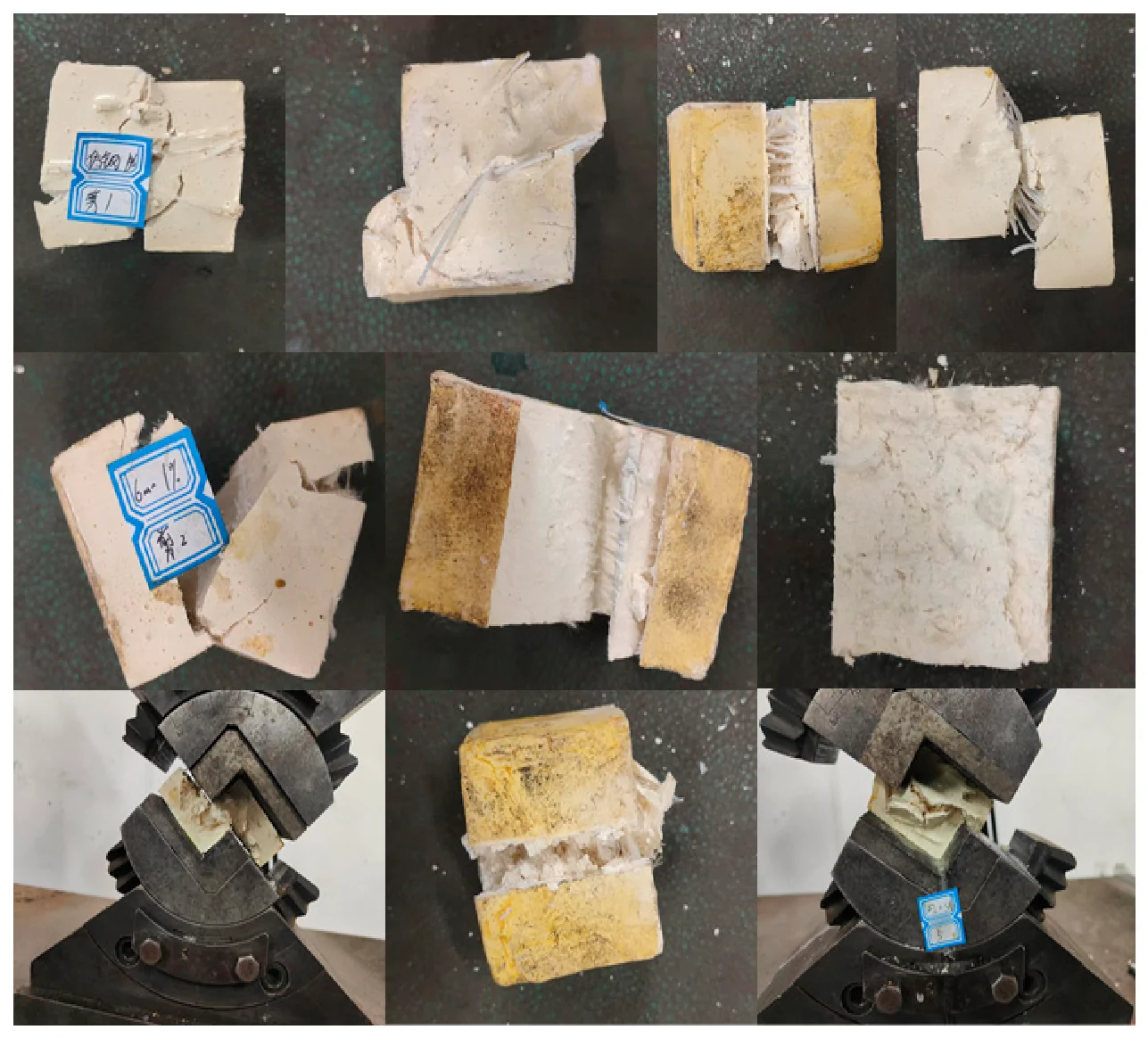
Shear specimens after testing.

For the TPPF-reinforced groups, TPPF-S-0.75% achieved the highest cohesion of 8.65 MPa and an internal friction angle of 24.13°, indicating the best shear-resistance performance among the tested fiber-reinforced groups. Compared with the unmodified matrix, the increase in cohesion of the TPPF-S-0.75% group indicates that toughened polypropylene fibers can enhance the cohesive resistance and crack-bridging capacity of the TSL material under shear loading. Similar enhancement mechanisms have been observed in fiber-reinforced polymer composites, where toughened polypropylene fibers improve energy dissipation capacity and delay crack propagation by forming effective bridging networks within the matrix [30]. The shear failure modes of different specimens are shown in Figure 10.

As shown in Figure 10, the macroscopic morphology of the shear failure surfaces varied with fiber type and fiber content, changing from relatively smooth to rougher and more undulating surfaces [28,31]. The specimens reinforced with toughened polypropylene fibers generally exhibited more irregular cracks and more uneven shear surfaces after failure. At the macroscopic scale, this observation may suggest a stronger resistance to crack propagation during shear failure [13,28,31]. The shear surfaces of the PVA-reinforced groups appeared relatively more uniform, which may indicate a more stable interaction between the fibers and the matrix under the present test conditions. In contrast, the PPMF-reinforced groups showed relatively less stable shear-surface morphology. However, it should be noted that these interpretations are based mainly on macroscopic post-failure observations rather than direct microscopic evidence. Therefore, the possible roles of fiber–matrix interaction, crack-bridging behavior, and local fiber distribution during shear failure require further investigation by microscopic observation and quantitative image analysis.

#### 2.2.3. Tensile Test Results and Analysis

For clarity and consistency with the compression and variable-angle shear tests, the tensile-test groups were uniformly coded as Fiber–T–Content, where Fiber denotes the fiber type, T denotes the tensile test, and Content denotes the fiber volume fraction. In this coding system, PVA, PPMF, and TPPF represent polyvinyl alcohol fiber, polypropylene mesh fiber, and toughened polypropylene fiber, respectively, while M represents the unmodified matrix material. For example, PVA-T-1.00% represents the PVA-fiber-reinforced polymer-based TSL material with a fiber volume fraction of 1.00% under tensile testing, whereas M-T-0% represents the unmodified matrix material under tensile testing.

**Figure 11:**
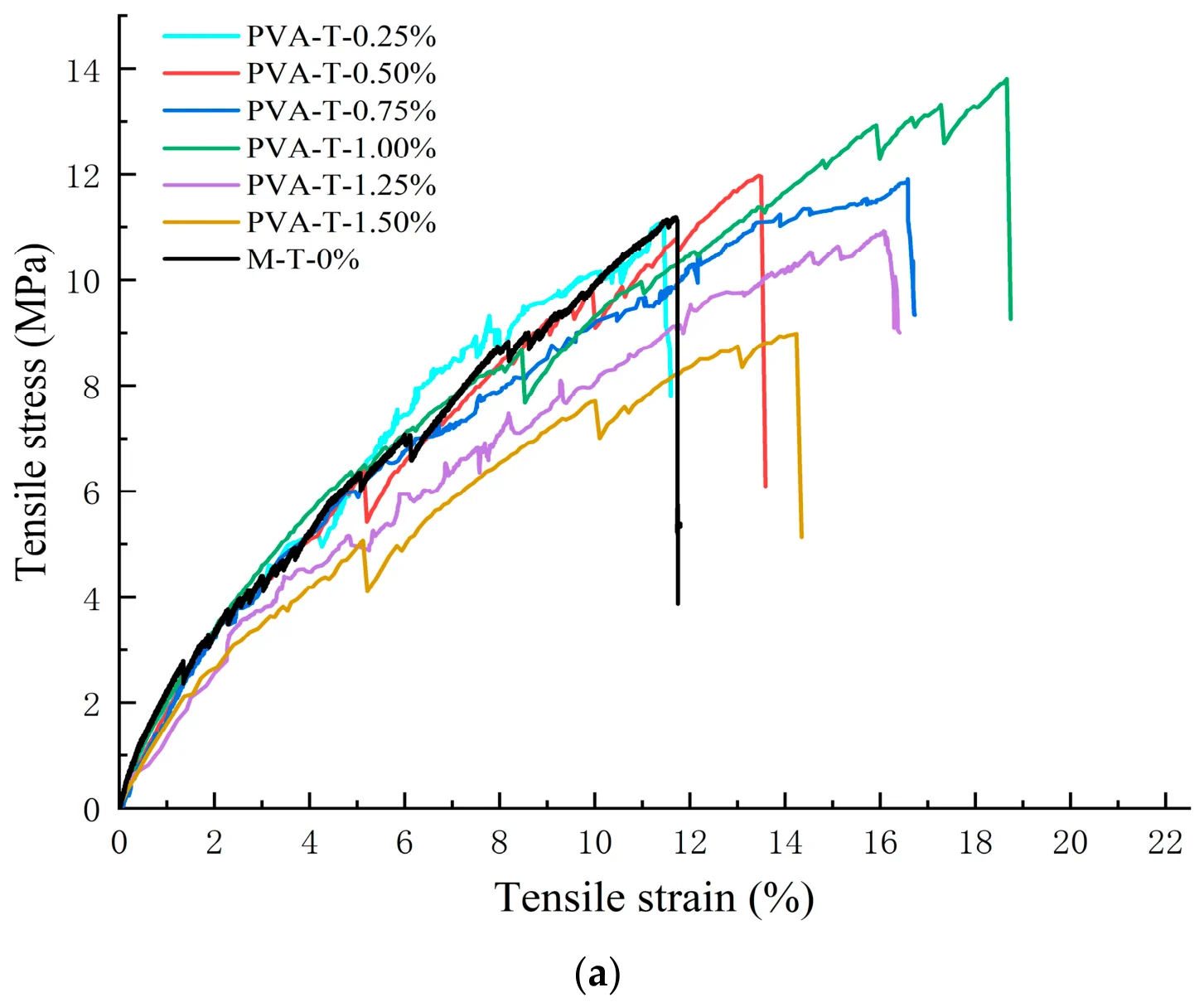

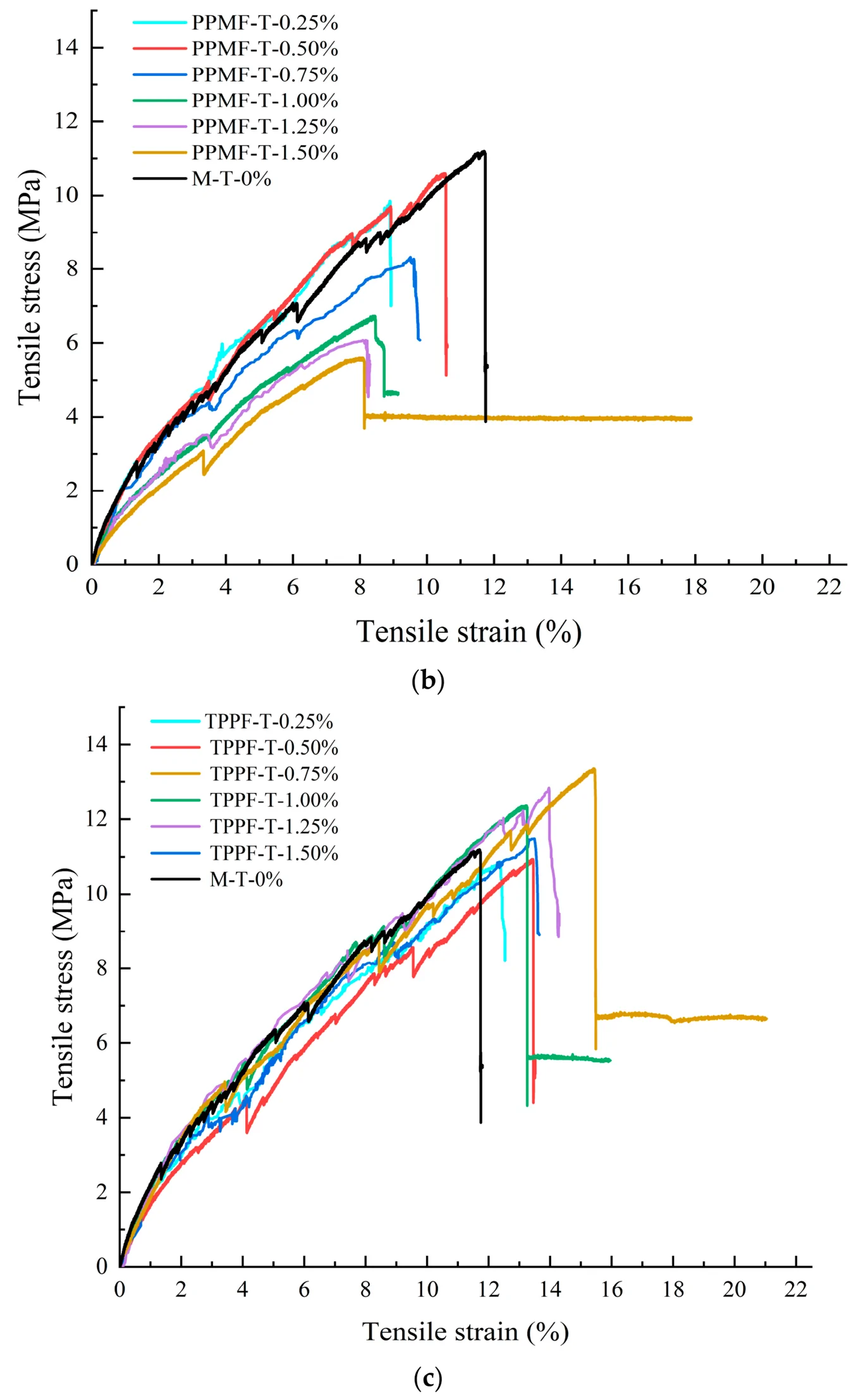
Tensile stress–strain curves of unmodified and fiber-reinforced polymer-based TSL materials: (**a**) PVA fiber-reinforced polymer-based TSL materials. (**b**) PPMF-reinforced polymer-based TSL materials. (**c**) TPPF-reinforced polymer-based TSL materials.

Figure 11 shows the tensile stress–strain curves of the unmodified and fiber-reinforced polymer-based TSL materials. To avoid overcrowding in the figure and to make the comparison among different fiber contents clearer, the curves are presented as representative averaged tensile responses for each group. The stress–strain curves alone cannot fully reflect the tensile reinforcement effect of different fibers. Therefore, the key tensile parameters, including peak tensile stress, strain at peak stress, fracture/end strain, and tensile toughness to fracture, were further calculated from the tensile test data and summarized in Table 5. These parameters provide a quantitative basis for comparing the tensile strength, deformation capacity, and energy-absorption capacity of the different fiber-reinforced TSL materials.

The corresponding tensile parameters obtained from the stress–strain curves are listed in Table 5. Compared with Figure 11, Table 5 provides a clearer quantitative comparison of tensile strength, deformation capacity, and tensile energy absorption among the unmodified matrix and the fiber-reinforced TSL materials.

As shown in Figure 11 and Table 5, the unmodified matrix material (M-T-0%) exhibited a peak tensile stress of 10.46 ± 2.25 MPa, a strain at peak stress of 23.06 ± 0.79%, a fracture/end strain of 23.62 ± 0.36%, and a tensile toughness of 1.47 ± 0.35 MJ/m^3^. After fiber incorporation, the tensile response of the polymer-based TSL material changed noticeably with fiber type and content. For the PVA-reinforced groups, relatively low fiber contents showed higher peak tensile stress and tensile toughness, indicating that PVA fibers can effectively improve tensile resistance and deformation coordination. However, excessive PVA fiber addition reduced the peak tensile stress and tensile toughness, which may be related to reduced dispersion quality and the formation of local defects at higher fiber contents.

For the PPMF-reinforced groups, the peak tensile stress was generally lower than that of the PVA- and TPPF-reinforced groups, suggesting that polypropylene mesh fibers were less effective in improving the tensile resistance of the polymer-based TSL material. Although the fracture/end strain and tensile toughness increased at higher PPMF contents, the overall tensile strength remained limited. For the TPPF-reinforced groups, the tensile toughness increased with fiber content, and TPPF-T-1.50% reached a tensile toughness of 2.68 ± 0.41 MJ/m^3^, indicating improved post-peak deformation and tensile energy-absorption capacity.

Overall, PVA fibers showed higher tensile reinforcement efficiency at relatively low contents, whereas TPPF contributed more noticeably to deformation tolerance and tensile energy absorption at higher contents. Considering the tensile stress–strain response together with the compression and variable-angle shear results, PVA-T-1.00% and TPPF-T-0.75% were retained as candidate reinforcement schemes for the subsequent buffered shear and true triaxial tests.

**Figure 12:**
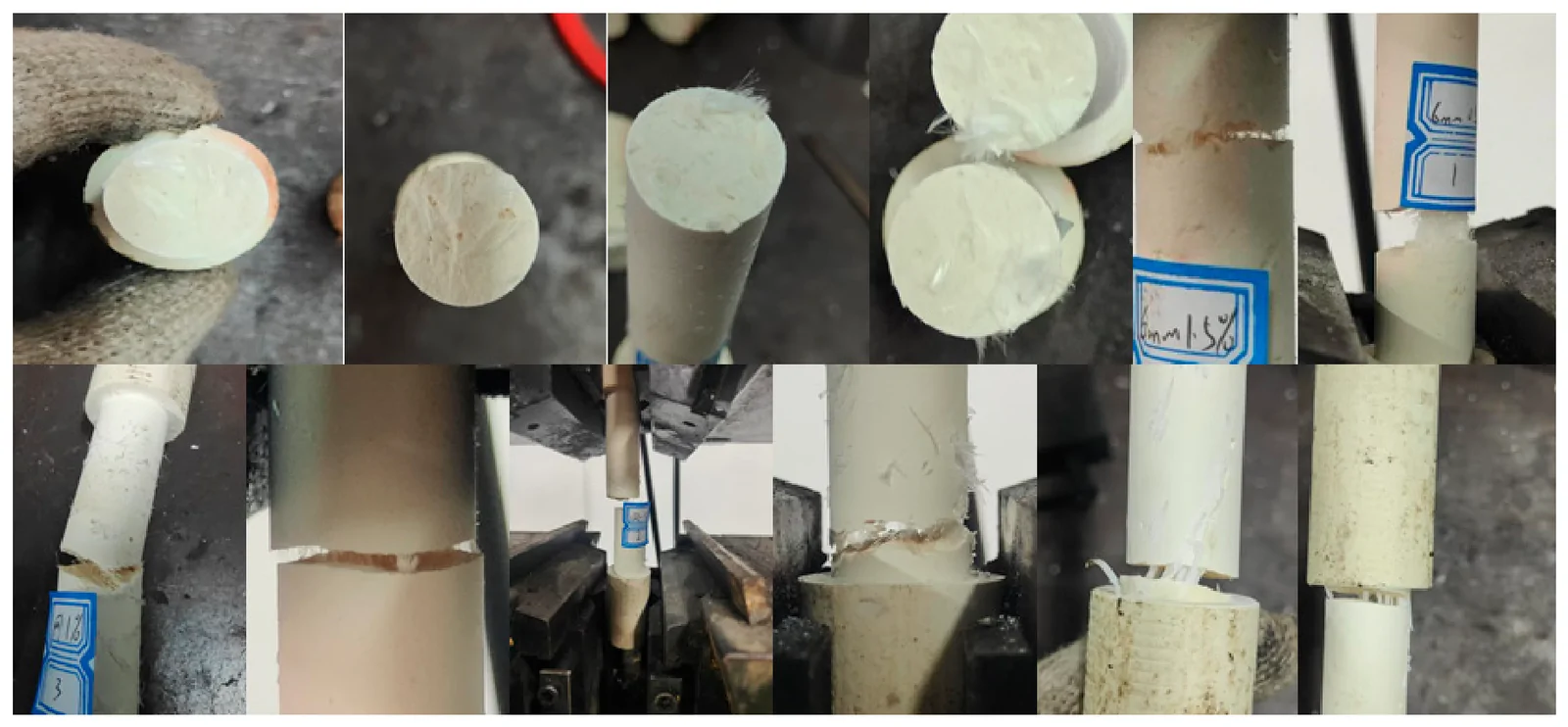
Failure modes of tensile specimens.

The tensile failure modes of the different specimens are shown in Figure 12. The unmodified matrix specimen showed a relatively rough fracture surface with visible pores, indicating that internal defects in the matrix may have influenced the tensile failure process. After fiber incorporation, the fracture morphology changed to varying degrees. The PVA-reinforced specimens generally showed more obvious filament-like features and better post-fracture integrity at the macroscopic scale, suggesting improved deformation coordination during tensile failure. The PPMF-reinforced specimens showed relatively irregular fracture surfaces, which may be associated with less uniform fiber distribution and limited tensile reinforcement efficiency. The TPPF-reinforced specimens exhibited more evident residual connection and surface roughness after failure, indicating that toughened polypropylene fibers may contribute to post-peak deformation resistance and energy dissipation. However, these interpretations are based mainly on macroscopic fracture morphology. Further SEM observation and quantitative fracture-surface analysis are required to clarify the detailed fiber–matrix interaction and crack-bridging mechanisms.

## 3. Spherical Indenter Buffering Test

### 3.1. Load-Bearing Basket Mechanism of TSL

**Figure 13:**
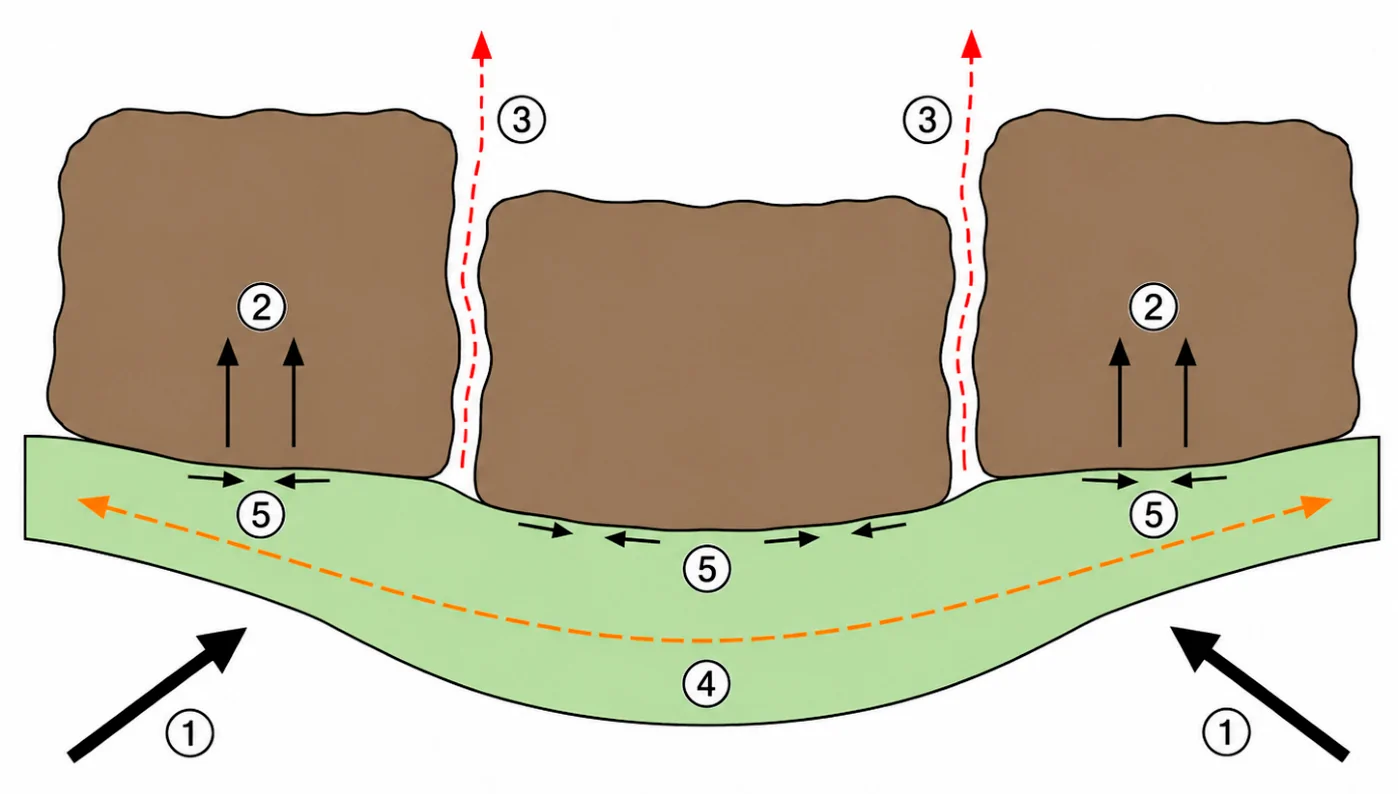
Load-bearing basket effect of TSL support.

When the surface of the tunnel surrounding rock is highly fractured or has experienced significant collapse, the rock and TSL form a bulge. The TSL and anchor rods work together to form a “load-bearing basket” [3,14,15,26] on the tunnel surface, as shown in Figure 13.

To clearly illustrate the mechanical behavior of the fiber-reinforced TSL system, the numbered annotations in Figure 13 are defined as follows:

1. Loading direction represents the externally applied load acting on the rock–TSL system. This loading induces overall deformation and governs the redistribution of stress within the structure.
2. Tensile stress zone indicates the region in the lower part of the rock mass where tensile stress is generated due to bending and differential displacement. This zone is considered the primary initiation area for crack development.
3. Crack propagation direction represents the preferential path of fracture growth, along which cracks propagate from the weak interface or defect regions toward the upper rock mass.
4. Tensile deformation zone refers to the deflected region of the TSL layer, where the material undergoes tensile bending deformation and contributes to energy dissipation.
5. Interfacial shear transfer and fiber reinforcement zone represents the contact region between the rock mass and the TSL layer, where interfacial shear stress is mobilized and fibers provide resistance to sliding, enhancing load transfer and structural stability.

Unlike loose rock fragments that have fallen off, the rock blocks within the load-bearing basket possess a certain load-bearing capacity due to compression from longitudinal and transverse stresses. This not only prevents the further free expansion of fragmented rock and fissures but also maintains the stress state of the deep rock mass as much as possible through the load transfer mechanism of the anchor bolts. The load-bearing basket mechanism of TSL involves three key influencing factors: First, during the formation of the load-bearing basket, the bending deformation properties and ductility of the TSL material determine the displacement of the TSL sprayed layer; second, after the load-bearing basket is formed, the tensile strength of the TSL material is the primary factor controlling the deformation of the load-bearing basket structure; and finally, fiber-reinforced TSL can enhance the load-bearing capacity of the load-bearing basket [10,13,29,30]. Considering the stress state of TSL within the load-bearing basket, this study employs buffered shear tests to further analyze the support performance of two types of fiber-reinforced TSL.

### 3.2. Materials and Methods for the Buffered Shear Test

Yilmaz [9] and Qiao et al. [27,32] have widely applied punch or indentation methods to evaluate the mechanical response of TSL materials, typically using a rigid punch and steel ring to characterize shear-dominated failure. However, such configurations mainly represent simplified interfacial shear conditions and do not fully capture the coupled deformation behavior of sprayed liners under complex ground support conditions.

In this study, a 50 mm diameter hemispherical indenter was adopted to better simulate the stress state of TSL layers under underground excavation conditions. The selected indenter size provides a representative contact scale for fractured rock blocks, while its curved geometry induces coupled compressive, tensile, and shear stresses, more closely reflecting stress redistribution caused by block movement and roof convergence. Compared with conventional flat punch or ring-shear tests, this method enables a more realistic evaluation of deformation coordination, crack evolution, and energy absorption capacity under combined loading. From an engineering perspective, this approach better reflects the interaction between sprayed liners and irregular rock blocks in roadway environments. However, the laboratory configuration still simplifies in situ conditions, particularly stress heterogeneity, confinement effects, and time-dependent behavior.

#### 3.2.1. Specimen Preparation

**Figure 14:**
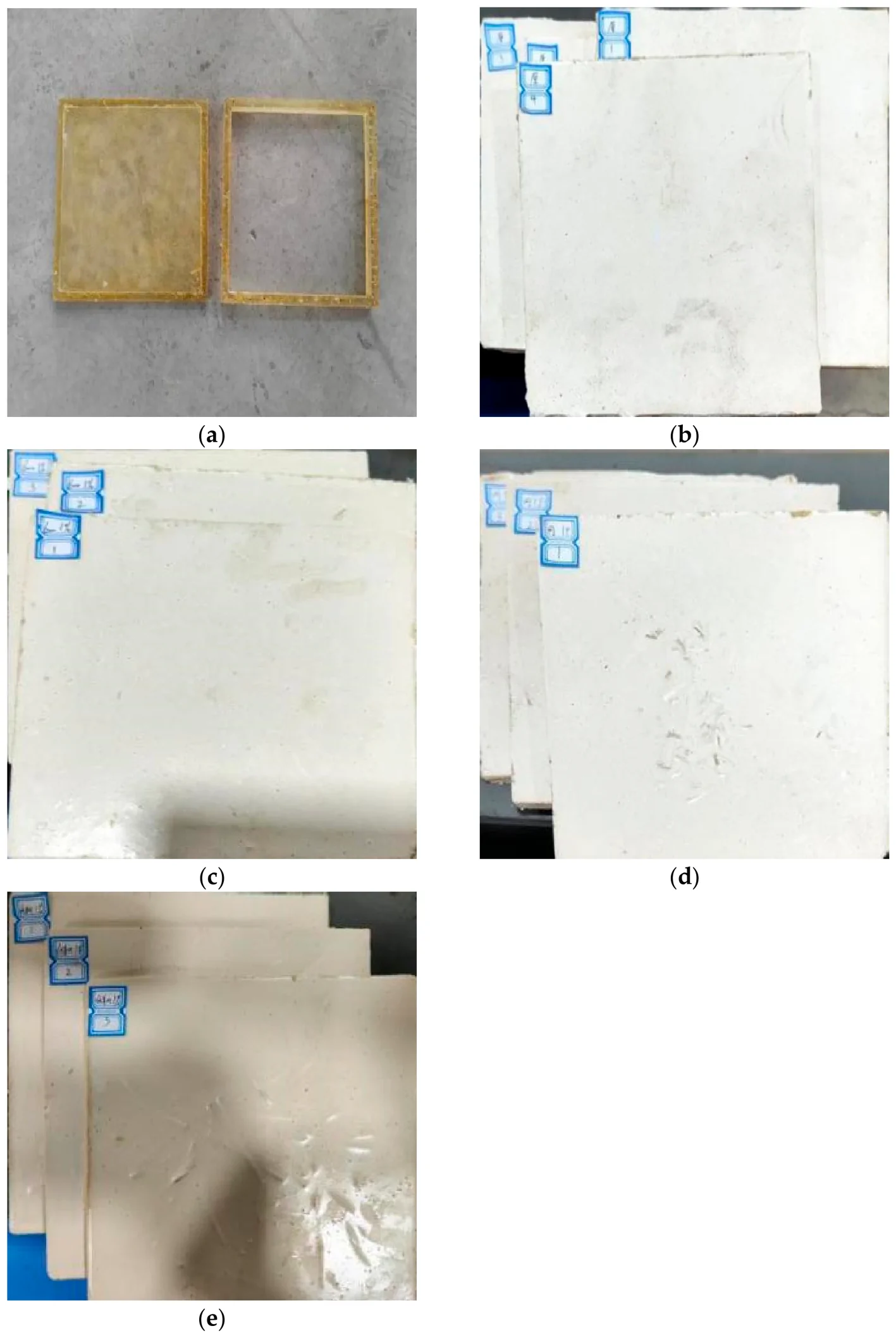
Acrylic mold plate to make specimens. (**a**) Plate-shaped test mold. (**b**) Raw material plate-shaped test specimen. (**c**) Polyvinyl alcohol fiber plate-shaped test specimen. (**d**) Mesh polypropylene fiber plate-shaped specimen. (**e**) Toughened polypropylene fiber plate-shaped specimen.

The 200 mm × 200 mm plate-shaped specimens were fabricated using acrylic plate molds, as shown in Figure 14. The mold contact surfaces were lubricated to facilitate demolding and reduce boundary adhesion effects.

The two-component polymer-based TSL material was mixed at a 1:1 volume ratio using a mechanical mixer and then transferred into a measuring container. The mixture was further stirred to obtain a uniform consistency before being poured into the acrylic molds, where it was allowed to spread evenly and fill the mold completely.

The material developed sufficient early strength, the specimens were demolded, and excess material was trimmed. Owing to the mild reaction, low heat release, and rapid early-strength development of the polymer-based TSL material, the specimens could be demolded approximately 2 min after casting. All demolded specimens were then conditioned at room temperature for 7 days before mechanical testing. For each material group, three identical specimens were prepared. The thickness of the plate-shaped specimens was controlled at 5 mm and maintained consistently across all groups. This thickness was selected to represent the typical scale of thin spray-on liner layers in underground support applications and to reduce size-dependent differences in stiffness, load-bearing capacity, and energy absorption behavior. Therefore, the comparative results mainly reflect the effect of fiber modification under the same plate geometry and curing condition.

#### 3.2.2. Experimental Procedure

**Figure 15:**
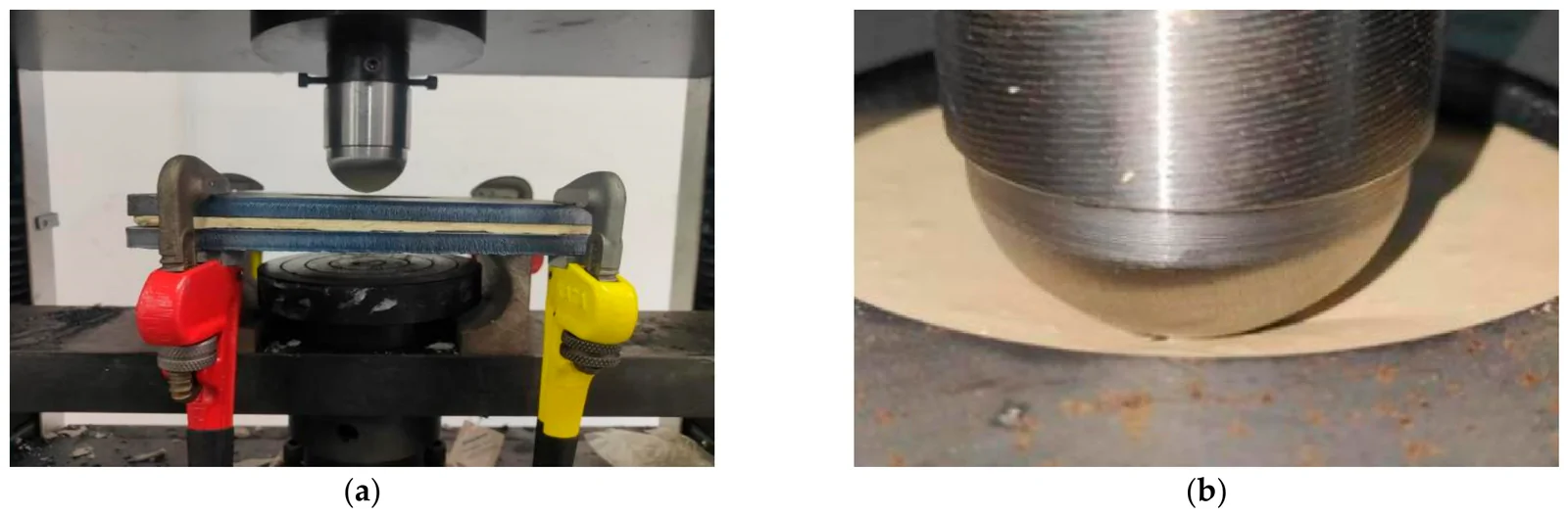
Buffer shear test. (**a**) Hydraulic press. (**b**) Specimen preparation.

The circular-indenter buffered shear test was conducted using a WAW-600C microcomputer-controlled electro-hydraulic servo universal testing machine, as shown in Figure 15a. A displacement-controlled loading mode was adopted at a loading rate of 1.5 mm/min. The loading device consisted of a 50 mm diameter hemispherical indenter, a rigid support plate with a central circular opening, and a clamping system. The plate-shaped TSL specimen had dimensions of 200 mm × 200 mm × 5 mm and was placed on the support plate with a 70 mm diameter central opening. The hemispherical indenter was vertically aligned with the center of the specimen and brought into direct contact with the upper surface of the TSL plate [31]. During loading, the indenter pressed the specimen into the unsupported central opening, producing a coupled stress state involving local compression beneath the indenter, tensile deformation in the spanning region, and shear stress near the edge of the opening. This testing configuration is commonly used to evaluate the deformation and energy absorption characteristics of thin plate materials under localized loading conditions [33].

To maintain consistent boundary conditions, the four corners of each specimen were fixed using pipe clamps, while the central lower region remained unsupported to allow downward deflection and failure observation, as shown in Figure 15b. For each material group, three parallel specimens were tested under the same loading and boundary conditions. During the test, the load and displacement data were continuously recorded by the testing system. The absorbed energy at an indenter displacement of 10 mm was calculated as the area under the load–displacement curve from the beginning of loading to a displacement of 10 mm:

(7) E10=∫010 Fδ dδ

where $E_{10}$ is the absorbed energy at an indenter displacement of 10 mm, F(δ) is the recorded load, and $E_{10}$ is the indenter displacement. For the discrete load–displacement data recorded by the testing system, $E_{10}$ was calculated using the trapezoidal rule:

(8) $$E_{10}=\overset{n-1}{\underset{i=1}{\sum }}\frac{\left(F_{i}+F_{i+1}\right)}{2}\left(\delta_{i+1}-\delta_{i}\right)$$

This equation was implemented in Origin 2021 software using the trapezoidal integration method to calculate the absorbed energy from the discrete load–displacement data.

Where $F_{i}$ and $F_{i+1}$ are adjacent load values, and $\delta_{i}$ and $\delta_{i+1}$ are the corresponding displacement values. When the load is expressed in kN and the displacement is expressed in mm, the calculated energy is expressed in J. The fixed displacement interval of 10 mm was adopted to ensure a consistent comparison of energy absorption capacity among different fiber-reinforced TSL materials.

### 3.3. Results and Analysis

**Figure 16:**
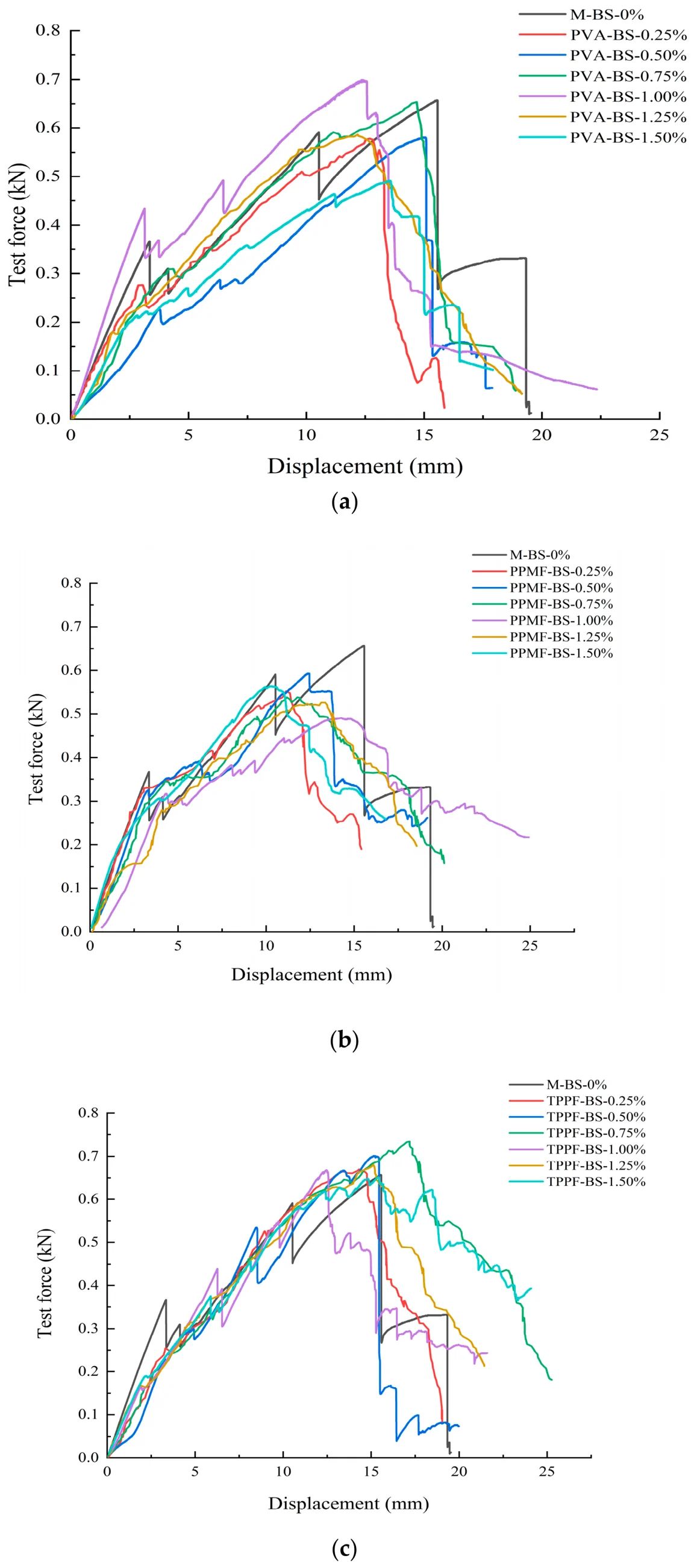
Load–displacement curves of unmodified and fiber-reinforced TSL plates in the circular-indenter buffered shear test. (**a**) PVA-reinforced. (**b**) PPMF-reinforced. (**c**) TPPF-reinforced.

The experimental results of the circular-indenter buffered shear test for specimens with different fiber types and fiber contents are shown in Figure 16 and Table 6. To ensure consistency among the text, figure legends, and table entries, the material groups were uniformly coded as Fiber–BS–Content. In this coding system, Fiber denotes the fiber type, BS denotes the circular-indenter buffered shear test, and Content denotes the fiber volume fraction. M represents the unmodified matrix material, while PVA, PPMF, and TPPF represent polyvinyl alcohol fiber, polypropylene mesh fiber, and toughened polypropylene fiber, respectively. For example, PVA-BS-1.00% represents the PVA-fiber-reinforced polymer-based TSL plate with a fiber volume fraction of 1.00% in the buffered shear test, whereas M-BS-0% represents the unmodified matrix material without fiber addition.

For each material group, three plate-shaped parallel specimens were tested under the same loading and boundary conditions. The load–displacement curves presented in Figure 16 represent the arithmetic mean responses of the valid specimens in each group. To further quantify the repeatability and dispersion of the buffered shear results, Table 6 summarizes the corresponding quantitative parameters, including peak load, displacement at peak load, load at 10 mm displacement, and absorbed energy at 10 mm displacement, expressed as mean ± standard deviation. These parameters were selected because the support performance of TSL materials should be evaluated not only by conventional strength indices, but also by load–displacement response, deformation capacity, load-retention behavior, and energy absorption capacity [14,26,33].

The unmodified matrix specimen (M-BS-0%) exhibited a peak load of 0.694 ± 0.052 kN, with a displacement at peak load of 14.658 ± 1.100 mm and an absorbed energy of 3.263 ± 0.075 J within the displacement interval of 0–10 mm. After fiber incorporation, the buffered shear response varied with fiber type and fiber content. Compared with Figure 16 alone, the statistical results in Table 6 provide a clearer basis for comparing the load-bearing capacity, deformation response, and energy absorption performance of the unmodified and fiber-reinforced polymer-based TSL plates.

To further quantify the differences observed in the load–displacement curves, the peak load, displacement at peak load, load at 10 mm displacement, and absorbed energy at 10 mm displacement were extracted from the original test data and are summarized in Table 6 as mean ± standard deviation.

As shown in Figure 16 and Table 6, the circular-indenter buffered shear response varied with fiber type and fiber content. The unmodified matrix specimen (M-BS-0%) exhibited a peak load of 0.694 ± 0.052 kN, a displacement at peak load of 14.658 ± 1.100 mm, and an absorbed energy of 3.263 ± 0.075 J within the displacement interval of 0–10 mm. For the PVA-reinforced groups, the peak load first increased and then decreased with increasing fiber content, with PVA-BS-1.00% showing a relatively high peak load of 0.636 ± 0.055 kN and an absorbed energy of 3.118 ± 0.832 J. This indicates that an appropriate PVA fiber content can improve the load-bearing response and deformation coordination of the polymer-based TSL plate under coupled compression–tension–shear loading. For the PPMF-reinforced groups, the peak load was generally lower than that of the unmodified matrix and the other fiber-reinforced groups, indicating that polypropylene mesh fibers were less effective in improving the buffered shear bearing capacity. In contrast, the TPPF-reinforced groups showed an overall increase in load at 10 mm displacement and absorbed energy with increasing fiber content [26,34], suggesting improved load-retention behavior and energy absorption capacity during the buffered shear process.

**Figure 17:**
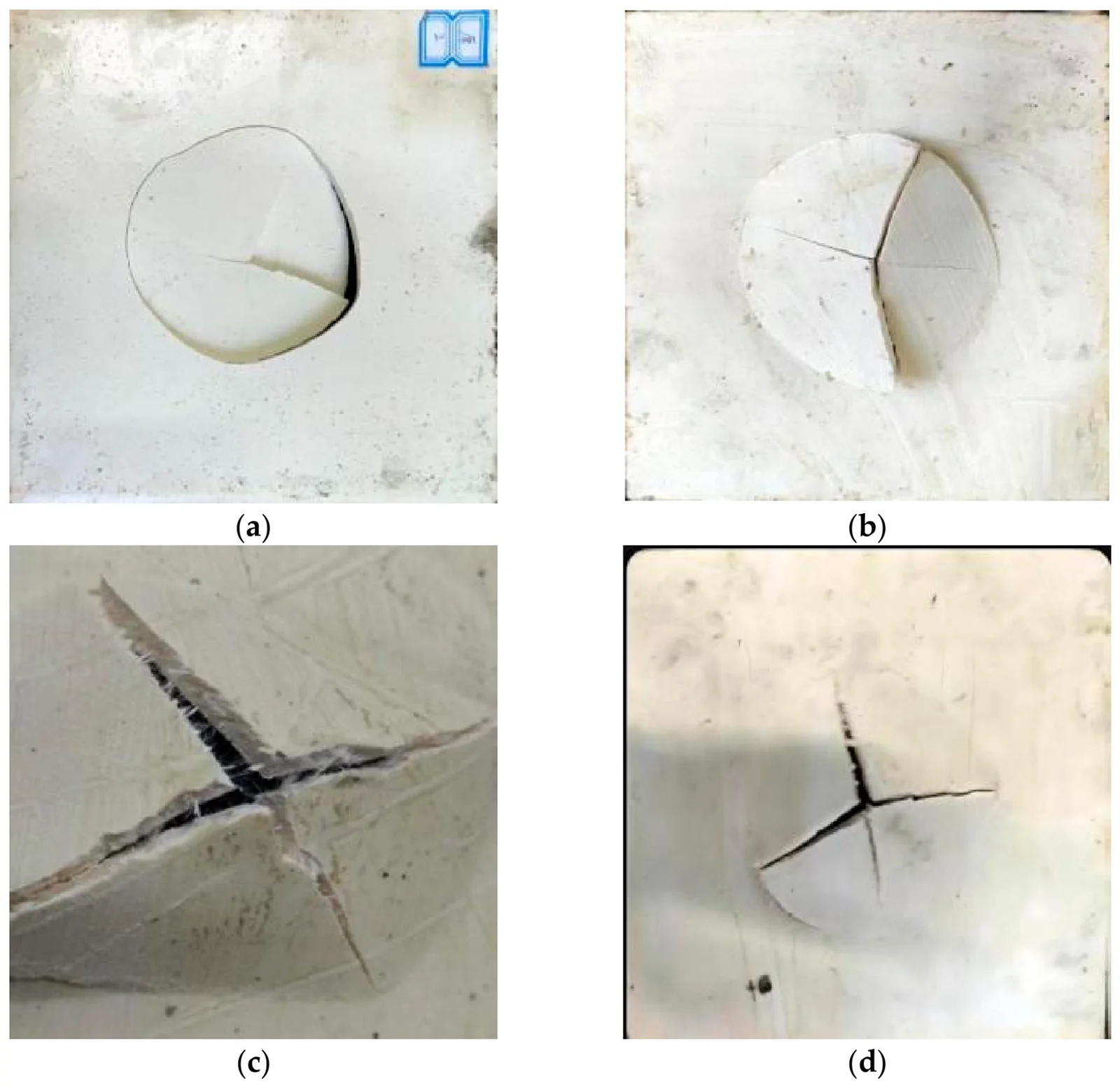
Failure modes of different plate specimens. (**a**) After failure of the raw material board specimen. (**b**) After failure of the polyvinyl alcohol fiberboard specimen. (**c**) After failure of the polypropylene mesh fiberboard specimen. (**d**) Toughened polypropylene fiberboard specimen.

The failure patterns of different plate-shaped specimens are shown in Figure 17. In the buffered shear test, the specimens exhibited typical tensile-shear composite failure: in the initial stage, localized indentation occurred in the area under the spherical indenter; subsequently, cracks propagated radially from the compressed zone; finally, the cracks extended to the vicinity of the clamping boundaries, with significant shear failure appearing in the edge regions [27,28,33]. Compared to the raw material, more exposed fibers can be observed in the fiber-modified specimens during crack propagation. This indicates that fiber bridging delays rapid crack penetration [13,31], reduces stress concentration in the edge zone, and allows the specimen to retain good integrity and residual load-bearing capacity even after failure.

Based on the results of the basic mechanical tests and the circular-indenter buffered shear tests, the PVA-1.00% and TPPF-0.75% groups were selected as candidate reinforcement schemes for the subsequent true triaxial tests, as summarized in Table 7. The PVA-1.00% group was selected because it showed favorable tensile reinforcement, deformation coordination, and stable buffered shear response. The TPPF-0.75% group was selected because it exhibited superior shear resistance in the variable-angle shear test and good load-bearing behavior in the buffered shear test. Therefore, these two groups were further compared under true triaxial loading conditions to determine the optimal fiber-reinforcement scheme for polymer-based TSL materials.

## 4. True Triaxial Tests

### 4.1. Test Principle and Stress Conditions

During the stress redistribution process, if the load on the shallow surrounding rock exceeds its strength, the rock mass undergoes plastic deformation or even fracturing, transferring the load to the deeper rock mass [14,26]. At this point, the TSL and the bonded surrounding rock form a composite structure that jointly bears the load and deforms. The TSL lining establishes a constraint layer for the internal surrounding rock through its own load-bearing capacity, with its primary support function being to constrain the deformation of the deeper surrounding rock.

Shotcrete provides a “shell-like” surface support function, and TSL, when bonded to the rock mass, can provide the same support effect. Although TSL may experience localized delamination, distortion, tearing, or even leakage, the main body remains bonded to the rock mass. TSL and the bonded rock mass, including the rock blocks within the load-bearing zone, can form a thin-shell structure to bear the load.

In summary, TSL alters the stress state of the bonded rock mass through its own load-bearing capacity, thereby constraining the deformation of the surrounding rock. Simultaneously, TSL and the bonded rock mass form a thin-shell structure to bear the load [26]. Based on the aforementioned stress environment of TSL, this study employs true triaxial tests with a single open face and lateral confining pressure to compare and analyze the load-bearing performance of two types of fiber-reinforced TSL and determine the optimal fiber-reinforcement scheme.

### 4.2. Materials and Methods for the True Triaxial Test

To further compare the reinforcing effects of the 1.0% PVA fiber and 0.75% TPPF groups under a more representative engineering stress condition, true triaxial compression tests were performed. In underground roadways, TSL materials are subjected to complex three-dimensional stress redistribution induced by excavation, surrounding-rock deformation, and localized compression–shear interactions. Therefore, the true triaxial test was adopted to reproduce a controlled three-dimensional stress state and to evaluate the deformation resistance and energy-dissipation characteristics of the two selected fiber-reinforced TSL materials under different principal stress conditions.

**Figure 18:**
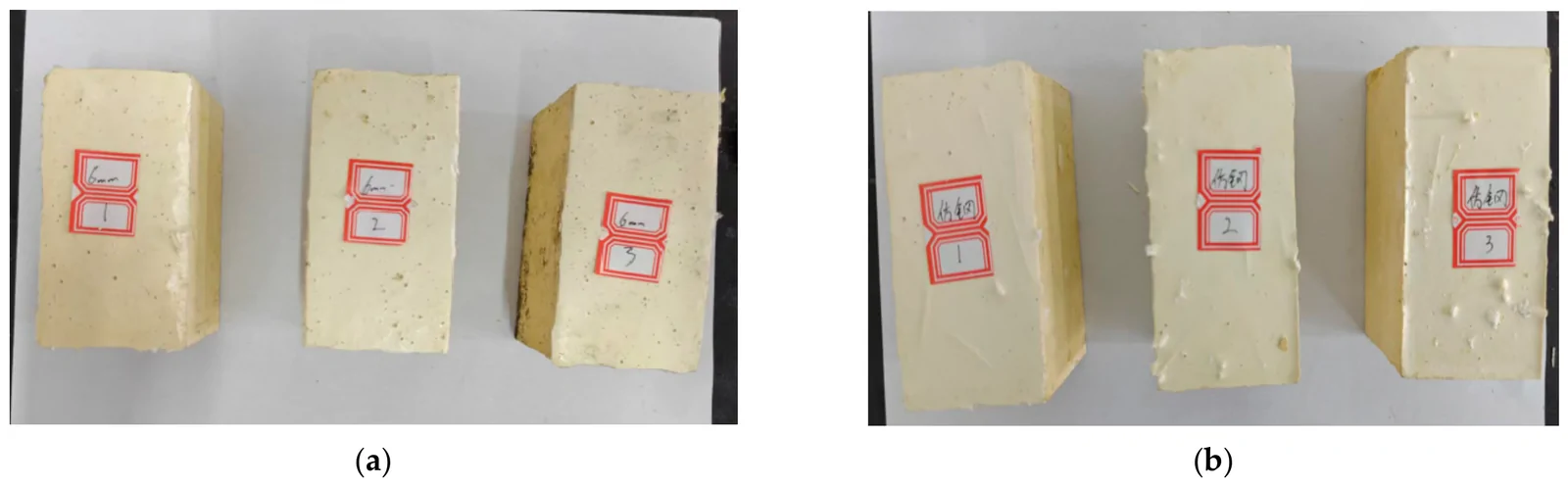
Specimen fabrication. (**a**) Preparation of thin sprayed-on layer specimens containing 1% polyvinyl alcohol fibers. (**b**) Preparation of thin sprayed-layer specimens containing 0.75% toughened polypropylene fibers.

According to the Test Methods for Coal and Rock Mechanics, prismatic specimens with dimensions of 50 mm × 50 mm × 100 mm were prepared for the true triaxial compression tests, as shown in Figure 18. Although polymer-based TSL materials are typically applied in the field as thin layers with a thickness of approximately 5–8 mm, the standardized specimen geometry was used in this study to satisfy the loading requirements of the true triaxial testing system, ensure stable contact between the specimen and loading platens, and obtain a relatively uniform three-dimensional stress distribution during testing. Prior to testing, the loading surfaces of all specimens were carefully polished to minimize end friction and reduce stress concentration caused by surface irregularities.

**Figure 19:**
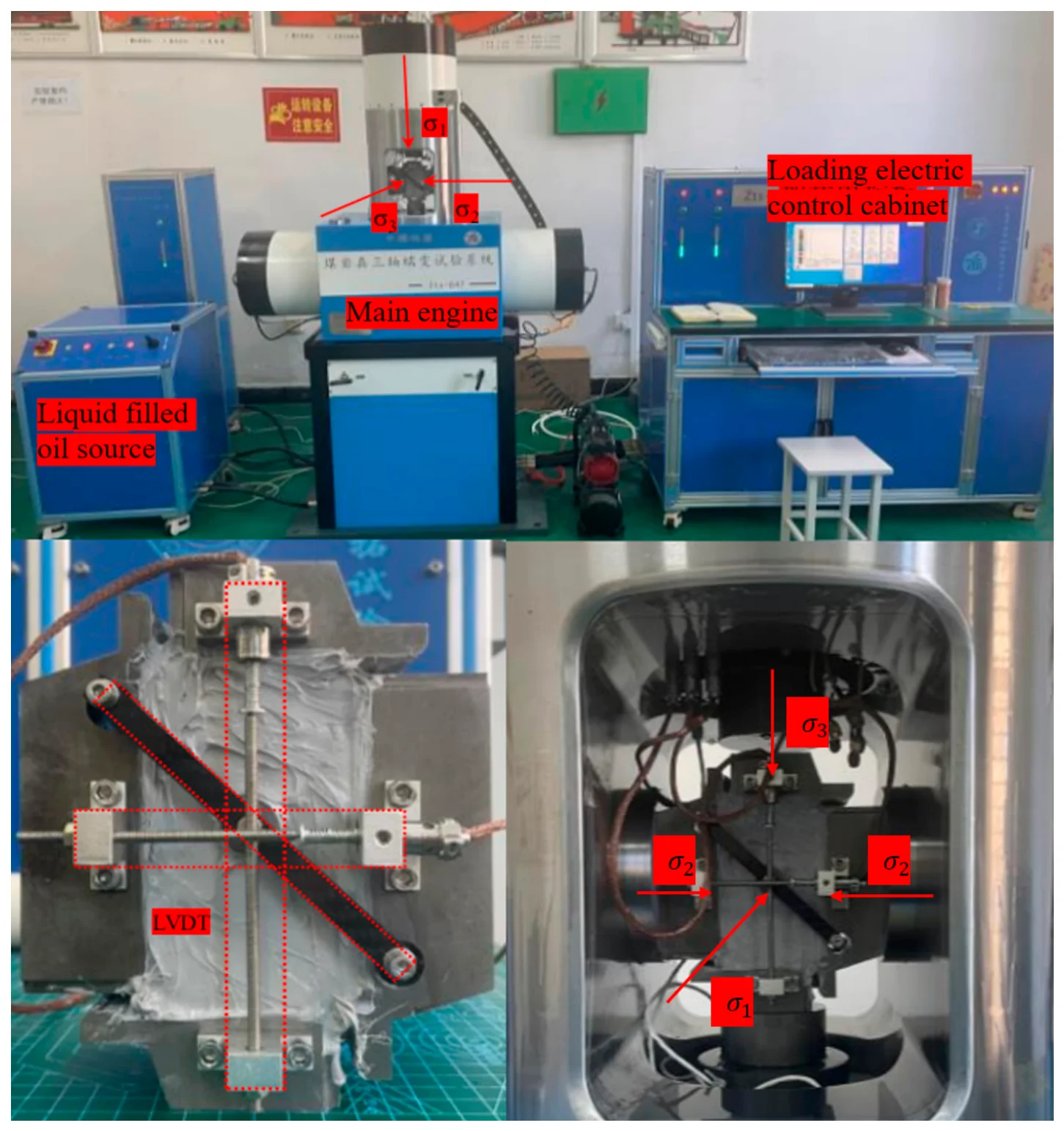
Coal-rock true triaxial test system.

All triaxial tests were conducted using the ZTS-847 coal rock true triaxial testing system. The entire system consists of three components: the main unit, the loading control cabinet, and the fluid supply unit, as shown in Figure 19. Prior to testing, a friction reducer was applied to the grips and copper foil to reduce friction between the specimen and the indenter, and the front and back surfaces of the specimen were wrapped with sealant to prevent specimen fragmentation and contamination of the fluid source.

A “two-rigid, one-flexible” loading method was employed, where the σ_1_ and σ_2_ directions were loaded using rigid metal indenters, while the σ_3_ direction was loaded hydraulically. Three LVDT displacement sensors were used to monitor and record the specimen’s deformation in the three stress directions in real time during the experiment.

Lateral confinement (σ_2_): A constant hydrostatic pressure of 1 MPa is applied to both left and right sides of the specimen. This pressure remains constant, simulating the constant confining pressure provided by the surrounding rock in the axial direction of the tunnel.

Axial loading (σ_1_): A displacement-controlled or stress-controlled compressive load is applied along the longitudinal axis of the specimen (simulating the normal direction to the tunnel surface) until the specimen undergoes significant failure or reaches a predetermined displacement. This direction simulates the deformation caused by tunnel confining pressure.

Free surface (σ_3_): No active load is applied to the front and rear faces of the specimen; the front face is maintained as a free surface, simulating the unconfined face of the TSL support in the tunnel, while the rear face is constrained by a stop plate, representing the rock mass bonded to the TSL.

Data Acquisition: The system records axial load, axial displacement, and lateral displacement (optional) in real time, and plots stress–strain curves based on these measurements.

### 4.3. Results and Analysis

**Figure 20:**
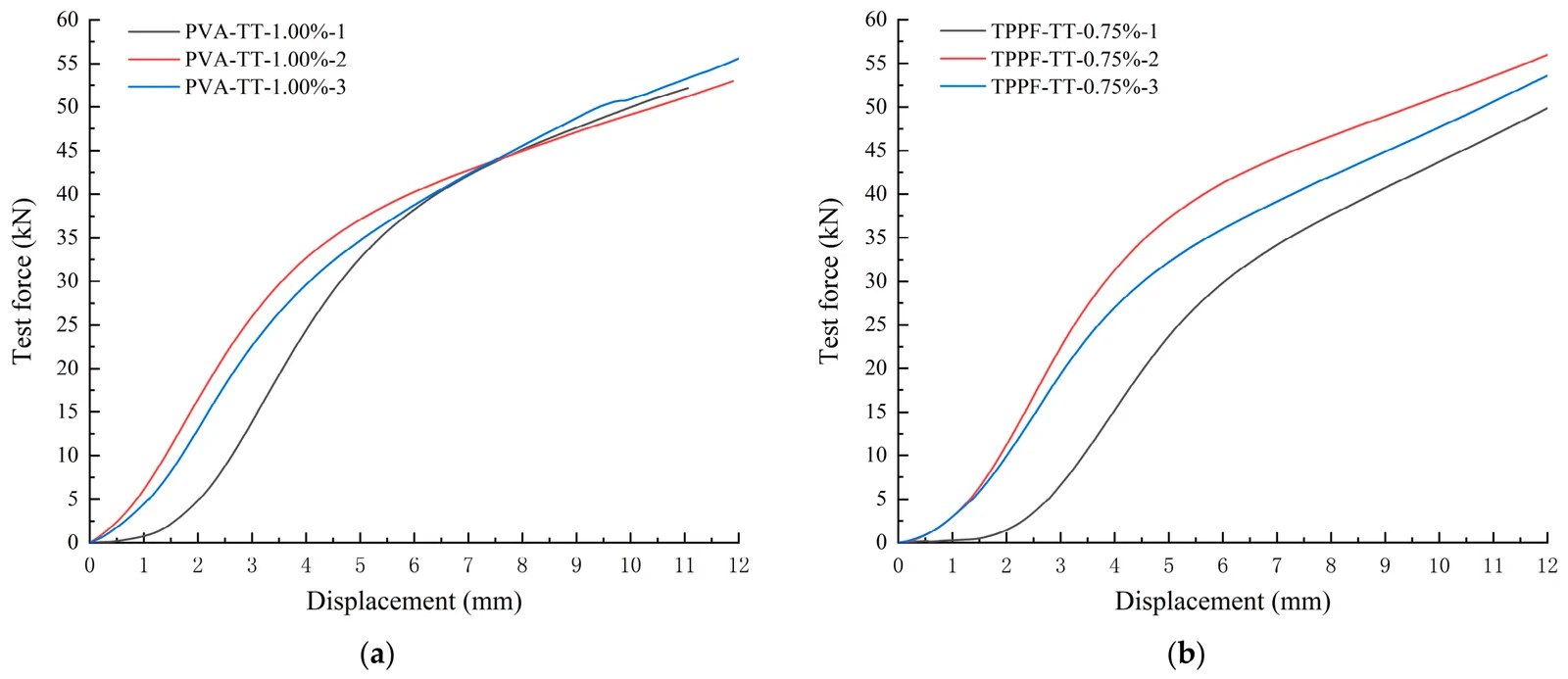
Triaxial test force–displacement curves. (**a**) Test force versus displacement curve for toughened polypropylene fiber modification. (**b**) Test force versus displacement plot for polyvinyl alcohol fiber modification.

The load–displacement curves for the 1.0% polyvinyl alcohol fiber group and the 0.75% toughened polypropylene fiber group are shown in Figure 20. As shown in Figure 20, the overall shapes of the two curves are similar, both exhibiting characteristics of rapid load increase in the early stage [26,28], a slowing slope in the middle stage, and continued stable growth in the late stage. At a displacement of approximately 5 mm, the test forces for all three groups had already exceeded 30 kN; at a displacement of approximately 10 mm, the test forces for the 1.0% polyvinyl alcohol fiber group and the 0.75% toughened polypropylene fiber group were approximately 51, 48, and 50 kN, respectively, with no significant difference, indicating that the two groups are generally comparable in terms of pre-peak load-bearing capacity.

In terms of energy absorption [26,34], the absorbed energy was calculated according to Equation (7). Specifically, the energy absorption at a displacement of 10 mm was obtained by integrating the area under the load–displacement curve over the displacement interval of 0–10 mm. The integration was performed using the original load–displacement data recorded during the true triaxial tests, and the discrete data were processed using the trapezoidal rule.

Based on this calculation, when the displacement reached 10 mm, the 1.0% polyvinyl alcohol (PVA) fiber group absorbed 311.4 J of energy, whereas the 0.75% toughened polypropylene fiber (TPPF) group absorbed 295.0 J. Compared with the 0.75% TPPF group, the 1.0% PVA fiber group exhibited an approximately 5.5% higher energy absorption capacity [26,34]. This indicates that although the 0.75% TPPF group showed better performance in individual mechanical indices, such as compressive strength and shear strength, the 1.0% PVA fiber group demonstrated superior load-bearing balance and energy-dissipation capacity under true triaxial conditions, which are closer to the complex three-dimensional stress environment of deep roadways [11,14,26]. Therefore, based on the combined results of the basic mechanical tests, buffered shear tests, and true triaxial tests, the 1.0% PVA fiber-reinforced polymer-based TSL material was identified as the preferred fiber-reinforcement scheme [13,26].

## 5. Conclusions

Through uniaxial compression, variable-angle shear, dowel tensile, buffered shear, and true triaxial tests [13,14,26,33], the effects of different fibers on the mechanical properties and support performance of polymer thin-spray liner materials were systematically investigated. The main conclusions are as follows:

1. The basic mechanical test results showed that toughened polypropylene fibers had a more significant reinforcing effect on the compressive and shear properties of the polymer thin-spray material [13,14]. In particular, the 0.75% toughened polypropylene fiber group exhibited an internal cohesion of 8.65 MPa and an internal friction angle of 24.12°. In comparison, polyvinyl alcohol fibers showed better performance in tensile resistance and deformation coordination [13,29]. The 1.0% PVA fiber group exhibited a compressive strength of approximately 49.87 MPa and a peak tensile stress close to 14 MPa. The overall reinforcing effect of polypropylene mesh fibers was relatively limited.
2. Considering the membrane-like load-bearing effect of TSL support [31], a circular indenter buffered shear test was conducted to evaluate the combined tensile–shear bearing capacity of the fiber-reinforced polymer thin-spray materials [33]. The results showed that both the 1.0% PVA fiber group and the 0.75% toughened polypropylene fiber group exhibited good tensile–shear load-bearing capacity. Among them, the 1.0% PVA fiber group showed more balanced comprehensive performance in terms of deformation coordination, bearing stability, and post-peak load-bearing behavior.
3. To further evaluate the reinforcement effects under a three-dimensional stress state similar to that of underground roadways, true triaxial tests were conducted on the 0.75% toughened polypropylene fiber group and the 1.0% PVA fiber group [26]. At a displacement of 10 mm, the energy absorption of the 1.0% PVA fiber group was approximately 5.5% higher than that of the 0.75% toughened polypropylene fiber group. Therefore, although the 0.75% toughened polypropylene fiber group showed advantages in some basic mechanical indexes, the 1.0% PVA fiber-modified polymer thin-spray material exhibited better overall load-bearing balance and energy absorption capacity. Accordingly, the 1.0% PVA fiber-modified polymer thin-spray material was determined to be the optimal fiber-reinforcement scheme in this study.

## Figures and Tables

**Table 1:** Parameters of Fiber Materials.

Fiber Type	Tensile Strength (MPa)	Diameter (μm)	Elongation at Break (%)	Modulus of Elasticity (MPa)
Polyvinyl Alcohol Fiber (PVA)	1892	20	12	36,100
Polypropylene Mesh Fiber (PPMF)	570	60	18	3550
Toughened Polypropylene Fiber (TPPF)	610	950	30	6100

**Table 2:** Peak compressive stress of parallel specimens for different fiber-reinforced TSL materials.

Group	Specimen 1/MPa	Specimen 2/MPa	Specimen 3/MPa	Mean ± SD/MPa
M-C-0%	51.76	52.03	52.21	52.00 ± 0.23
PVA-C-0.25%	44.18	44.49	44.74	44.47 ± 0.28
PVA-C-0.50%	42.46	42.71	43.02	42.73 ± 0.28
PVA-C-0.75%	44.08	44.35	44.59	44.34 ± 0.26
PVA-C-1.00%	49.66	49.84	50.11	49.87 ± 0.23
PVA-C-1.25%	46.54	46.69	46.81	46.68 ± 0.14
PVA-C-1.50%	40.37	40.54	40.68	40.53 ± 0.16
PPMF-C-0.25%	44.75	45.05	45.29	45.03 ± 0.27
PPMF-C-0.50%	39.94	40.13	40.29	40.12 ± 0.18
PPMF-C-0.75%	41.39	41.69	41.96	41.68 ± 0.29
PPMF-C-1.00%	49.31	49.57	49.74	49.54 ± 0.22
PPMF-C-1.25%	39.15	39.33	39.48	39.32 ± 0.17
PPMF-C-1.50%	37.24	37.51	37.69	37.48 ± 0.23
TPPF-C-0.25%	45.24	45.51	45.81	45.52 ± 0.29
TPPF-C-0.50%	49.85	50.15	50.39	50.13 ± 0.27
TPPF-C-0.75%	51.11	51.27	51.52	51.30 ± 0.21
TPPF-C-1.00%	48.48	48.75	48.93	48.72 ± 0.23
TPPF-C-1.25%	44.65	44.84	45.12	44.87 ± 0.24
TPPF-C-1.50%	48.12	48.47	48.73	48.44 ± 0.31

Note: C represents the uniaxial compression test. PVA, PPMF, and TPPF represent polyvinyl alcohol fiber, polypropylene mesh fiber, and toughened polypropylene fiber, respectively. Values are expressed as mean ± SD, *n* = 3.

**Table 3:** Shear test results of fiber-reinforced TSL materials.

Material	Shear Angle/°	Normal Stress/MPa	Shear Strength/MPa	Cohesion/MPa	Angle of Internal Friction/°
M-S-0%	42.00	22.90 ± 1.15	20.74 ± 1.04	6.02	33.60 ± 1.01
	50.00	11.29 ± 0.56	13.59 ± 0.68		
	58.00	7.36 ± 0.37	11.80 ± 0.59		
	66.00	3.51 ± 0.18	7.78 ± 0.39		
	74.00	2.06 ± 0.10	7.07 ± 0.35		
PVA-S-0.25%	42.00	28.98 ± 1.45	19.55 ± 0.98	7.55	23.50 ± 0.71
	50.00	18.16 ± 0.91	16.35 ± 0.82		
	58.00	10.54 ± 0.53	12.56 ± 0.63		
	66.00	6.23 ± 0.31	9.97 ± 0.50		
	74.00	3.92 ± 0.20	8.81 ± 0.44		
PVA-S-0.50%	42.00	31.58 ± 1.58	20.12 ± 1.01	7.76	21.33 ± 0.64
	50.00	18.42 ± 0.92	14.97 ± 0.75		
	58.00	10.53 ± 0.53	11.88 ± 0.59		
	66.00	7.89 ± 0.39	10.85 ± 0.54		
	74.00	5.26 ± 0.26	9.81 ± 0.49		
PVA-S-0.75%	42.00	26.46 ± 1.32	17.85 ± 0.89	8.12	20.66 ± 0.62
	50.00	16.62 ± 0.83	14.96 ± 0.75		
	58.00	9.64 ± 0.48	11.49 ± 0.57		
	66.00	6.47 ± 0.32	10.35 ± 0.52		
	74.00	4.41 ± 0.22	9.90 ± 0.50		
PVA-S-1.00%	42.00	30.08 ± 1.50	20.29 ± 1.01	8.45	22.27
	50.00	19.41 ± 0.97	17.48 ± 0.87		
	58.00	10.21 ± 0.51	12.16 ± 0.61		
	66.00	6.53 ± 0.33	10.45 ± 0.52		
	74.00	4.90 ± 0.25	11.00 ± 0.55		
PVA-S-1.25%	42.00	25.43 ± 1.27	17.16 ± 0.86	8.17	20.20
	50.00	16.50 ± 0.83	14.85 ± 0.74		
	58.00	9.92 ± 0.50	11.83 ± 0.59		
	66.00	6.74 ± 0.34	10.79 ± 0.54		
	74.00	4.15 ± 0.21	9.32 ± 0.47		
PVA-S-1.50%	42.00	28.56 ± 1.43	19.04 ± 0.95	7.87	21.34
	50.00	18.64 ± 0.93	15.15 ± 0.76		
	58.00	10.25 ± 0.51	11.88 ± 0.59		
	66.00	7.14 ± 0.36	10.67 ± 0.53		
	74.00	5.67 ± 0.28	10.10 ± 0.51		
PPMF-S-0.25%	42.00	18.76 ± 1.13	12.70 ± 0.76	6.41	18.62
	50.00	13.78 ± 0.83	11.06 ± 0.66		
	58.00	10.67 ± 0.64	9.99 ± 0.60		
	66.00	5.26 ± 0.32	8.06 ± 0.48		
	74.00	2.45 ± 0.15	7.23 ± 0.43		
PPMF-S-0.50%	42.00	31.58 ± 1.58	20.12 ± 1.01	7.76	21.33 ± 0.64
	50.00	18.42 ± 0.92	14.97 ± 0.75		
	58.00	10.53 ± 0.53	11.88 ± 0.59		
	66.00	7.89 ± 0.39	10.85 ± 0.54		
	74.00	5.26 ± 0.26	9.81 ± 0.49		
PPMF-S-0.75%	42.00	26.46 ± 1.32	17.85 ± 0.89	8.12	20.66 ± 0.62
	50.00	16.62 ± 0.83	14.96 ± 0.75		
	58.00	9.64 ± 0.48	11.49 ± 0.57		
	66.00	6.47 ± 0.32	10.35 ± 0.52		
	74.00	4.41 ± 0.22	9.90 ± 0.50		
PPMF-S-1.00%	42.00	17.91 ± 1.07	12.08 ± 0.72	6.47	21.09 ± 0.63
	50.00	15.19 ± 0.91	13.68 ± 0.82		
	58.00	8.54 ± 0.51	10.17 ± 0.61		
	66.00	5.57 ± 0.33	8.92 ± 0.54		
	74.00	3.07 ± 0.18	6.91 ± 0.41		
PPMF-S-1.25%	42.00	20.33 ± 1.22	13.71 ± 0.82	6.25	21.36 ± 0.64
	50.00	12.43 ± 0.75	11.19 ± 0.67		
	58.00	9.00 ± 0.54	10.72 ± 0.64		
	66.00	5.70 ± 0.34	9.12 ± 0.55		
	74.00	2.73 ± 0.16	6.14 ± 0.37		
PPMF-S-1.50%	42.00	17.85 ± 1.07	13.04 ± 0.78	6.18	21.02 ± 0.63
	50.00	14.26 ± 0.86	11.67 ± 0.70		
	58.00	7.38 ± 0.44	9.02 ± 0.54		
	66.00	5.72 ± 0.34	8.38 ± 0.50		
	74.00	2.67 ± 0.16	7.21 ± 0.43		
TPPF-S-0.25%	42.00	30.28 ± 1.82	20.42 ± 1.23	7.32	23.42 ± 0.70
	50.00	15.64 ± 0.94	14.08 ± 0.84		
	58.00	9.77 ± 0.59	11.64 ± 0.70		
	66.00	6.21 ± 0.37	9.94 ± 0.60		
	74.00	4.03 ± 0.24	9.06 ± 0.54		
TPPF-S-0.50%	42.00	30.00 ± 1.80	27.86 ± 1.67	7.94	23.67 ± 0.71
	50.00	16.26 ± 0.98	18.68 ± 1.12		
	58.00	10.16 ± 0.61	14.60 ± 0.88		
	66.00	7.11 ± 0.43	12.56 ± 0.75		
	74.00	4.05 ± 0.24	10.52 ± 0.63		
TPPF-S-0.75%	42.00	31.70 ± 1.90	21.38 ± 1.28	8.65	24.12 ± 0.72
	50.00	23.36 ± 1.40	21.04 ± 1.26		
	58.00	12.19 ± 0.73	14.52 ± 0.87		
	66.00	7.48 ± 0.45	11.97 ± 0.72		
	74.00	4.33 ± 0.26	9.72 ± 0.58		
TPPF-S-1.00%	42.00	30.45 ± 1.83	21.37 ± 1.28	8.06	23.73 ± 0.71
	50.00	21.45 ± 1.29	17.46 ± 1.05		
	58.00	12.56 ± 0.75	13.58 ± 0.81		
	66.00	7.34 ± 0.44	11.29 ± 0.68		
	74.00	5.67 ± 0.34	10.56 ± 0.63		
TPPF-S-1.25%	42.00	31.27 ± 1.88	21.09 ± 1.27	7.64	23.38 ± 0.70
	50.00	16.65 ± 1.00	14.99 ± 0.90		
	58.00	10.26 ± 0.62	12.23 ± 0.73		
	66.00	6.08 ± 0.36	9.74 ± 0.58		
	74.00	4.38 ± 0.26	9.83 ± 0.59		
TPPF-S-1.50%	42.00	29.98 ± 1.80	20.22 ± 1.21	7.50	24.71 ± 0.74
	50.00	21.25 ± 1.28	19.14 ± 1.15		
	58.00	8.87 ± 0.53	10.57 ± 0.63		
	66.00	6.87 ± 0.41	10.99 ± 0.66		
	74.00	4.15 ± 0.25	9.32 ± 0.56		

Note: Data are presented as mean ± standard deviation (*n* = 3). PVA, PPMF, and TPPF represent polyvinyl alcohol fiber, polypropylene mesh fiber, and toughened polypropylene fiber, respectively. M represents the unmodified matrix material, and S denotes the variable-angle shear test. σ and τ represent the normal stress and shear strength on the shear plane, respectively.

**Table 4:** Mohr–Coulomb fitting parameters of unmodified and fiber-reinforced polymer-based TSL materials.

Group	Fitting Equation	Cohesion c (MPa)	Internal Friction Angle φ (°)	R^2^
PVA-S-0.25%	τ = 7.55 + 0.435σ	7.55	23.51	0.957
PVA-S-0.50%	τ = 7.76 + 0.390σ	7.76	21.31	0.963
PVA-S-0.75%	τ = 8.12 + 0.377σ	8.12	20.66	0.952
PVA-S-1.00%	τ = 8.45 + 0.410σ	8.45	22.29	0.966
PVA-S-1.25%	τ = 8.17 + 0.368σ	8.17	20.20	0.958
PVA-S-1.50%	τ = 7.87 + 0.391σ	7.87	21.36	0.970
PPMF-S-0.25%	τ = 6.35 + 0.339σ	6.35	18.72	0.999
PPMF-S-0.50%	τ = 5.10 + 0.451σ	5.10	24.27	0.977
PPMF-S-0.75%	τ = 5.34 + 0.434σ	5.34	23.46	0.989
PPMF-S-1.00%	τ = 5.72 + 0.416σ	5.72	22.59	0.964
PPMF-S-1.25%	τ = 4.99 + 0.421σ	4.99	22.83	0.992
PPMF-S-1.50%	τ = 5.86 + 0.394σ	5.86	21.51	0.981
TPPF-S-0.25%	τ = 7.32 + 0.432σ	7.32	23.36	0.996
TPPF-S-0.50%	τ = 7.81 + 0.669σ	7.81	33.79	0.995
TPPF-S-0.75%	τ = 8.65 + 0.448σ	8.65	24.13	0.939
TPPF-S-1.00%	τ = 8.09 + 0.436σ	8.09	23.56	0.998
TPPF-S-1.25%	τ = 7.64 + 0.432σ	7.64	23.36	0.995
TPPF-S-1.50%	τ = 7.50 + 0.460σ	7.50	24.70	0.947
M-S-0%	τ = 6.02 + 0.664σ	6.02	33.58	0.974

Note: The Mohr–Coulomb fitting equation is expressed as $\tau_{s}=c+\sigma_{s}tan\left(\varphi \right)$, where τ is the shear strength, σ is the normal stress, c is the cohesion, and φ is the internal friction angle. The cohesion c corresponds to the intercept of the fitting equation, and the internal friction angle φ was calculated from the slope k using φ = arctan(k). R^2^ represents the coefficient of determination of the linear fitting. The shear angle used in the variable-angle shear test is different from the internal friction angle φ obtained from Mohr–Coulomb fitting.

**Table 5:** Tensile properties of unmodified and fiber-reinforced polymer-based TSL materials.

Group	Peak Tensile Stress (MPa)	Strain at Peak Stress (%)	Fracture/End Strain (%)	Tensile Toughness to Fracture (MJ/m^3^)
PVA-T-0.25%	13.61 ± 1.00	29.92 ± 9.55	60.87 ± 46.80	4.78 ± 3.26
PVA-T-0.50%	12.47 ± 1.19	28.44 ± 7.59	49.16 ± 32.45	3.71 ± 2.30
PVA-T-0.75%	11.34 ± 1.39	26.97 ± 5.63	37.45 ± 18.10	2.64 ± 1.34
PVA-T-1.00%	10.20 ± 1.58	25.49 ± 3.67	25.74 ± 3.75	1.57 ± 0.37
PVA-T-1.25%	9.60 ± 1.36	23.05 ± 3.95	23.59 ± 6.19	1.37 ± 0.44
PVA-T-1.50%	8.99 ± 1.13	20.60 ± 4.23	21.43 ± 8.63	1.16 ± 0.50
PPMF-T-0.25%	9.49 ± 2.70	17.96 ± 3.20	12.24 ± 2.45	0.77 ± 0.46
PPMF-T-0.50%	8.54 ± 1.95	17.37 ± 2.58	18.47 ± 2.18	0.96 ± 0.32
PPMF-T-0.75%	7.60 ± 1.20	16.78 ± 1.97	24.71 ± 1.91	1.16 ± 0.19
PPMF-T-1.00%	6.65 ± 0.45	16.19 ± 1.35	30.94 ± 1.64	1.35 ± 0.05
PPMF-T-1.25%	7.71 ± 2.47	18.23 ± 3.97	34.65 ± 2.90	1.85 ± 0.75
PPMF-T-1.50%	8.77 ± 4.48	20.26 ± 6.59	38.36 ± 4.16	2.34 ± 1.45
TPPF-T-0.25%	11.53 ± 0.18	20.43 ± 3.47	21.97 ± 5.80	1.53 ± 0.08
TPPF-T-0.50%	11.21 ± 0.78	22.30 ± 2.52	24.19 ± 4.76	1.60 ± 0.20
TPPF-T-0.75%	10.89 ± 1.38	24.17 ± 1.57	26.42 ± 3.72	1.67 ± 0.32
TPPF-T-1.00%	10.57 ± 1.98	26.04 ± 0.62	28.64 ± 2.68	1.74 ± 0.44
TPPF-T-1.25%	11.14 ± 1.71	26.59 ± 1.81	35.26 ± 2.67	2.21 ± 0.43
TPPF-T-1.50%	11.71 ± 1.43	27.13 ± 3.00	41.87 ± 2.65	2.68 ± 0.41
M-T-0%	10.46 ± 2.25	23.06 ± 0.79	23.62 ± 0.36	1.47 ± 0.35

**Table 6:** Buffered shear parameters of unmodified and fiber-reinforced TSL plates.

Group	Peak Load (kN)	Displacement at Peak Load (mm)	Load at 10 mm (kN)	Absorbed Energy at 10 mm, E10 (J)
M-BS-0%	0.694 ± 0.052	14.658 ± 1.100	0.559 ± 0.008	3.263 ± 0.075
PVA-BS-0.25%	0.587 ± 0.034	12.636 ± 1.387	0.483 ± 0.054	2.689 ± 0.535
PVA-BS-0.50%	0.603 ± 0.021	13.223 ± 1.645	0.490 ± 0.076	2.832 ± 0.630
PVA-BS-0.75%	0.620 ± 0.032	13.811 ± 1.928	0.496 ± 0.097	2.975 ± 0.729
PVA-BS-1.00%	0.636 ± 0.055	14.399 ± 2.228	0.503 ± 0.119	3.118 ± 0.832
PVA-BS-1.25%	0.610 ± 0.067	13.438 ± 0.527	0.509 ± 0.064	3.133 ± 0.450
PVA-BS-1.50%	0.583 ± 0.087	12.478 ± 1.524	0.514 ± 0.071	3.148 ± 0.429
PPMF-BS-0.25%	0.553 ± 0.092	12.938 ± 2.200	0.479 ± 0.077	3.178 ± 0.482
PPMF-BS-0.50%	0.545 ± 0.055	12.866 ± 0.850	0.467 ± 0.038	3.056 ± 0.262
PPMF-BS-0.75%	0.537 ± 0.025	12.794 ± 0.583	0.455 ± 0.027	2.935 ± 0.045
PPMF-BS-1.00%	0.529 ± 0.035	12.722 ± 1.922	0.443 ± 0.062	2.813 ± 0.179
PPMF-BS-1.25%	0.550 ± 0.002	11.622 ± 1.217	0.498 ± 0.050	3.152 ± 0.206
PPMF-BS-1.50%	0.572 ± 0.032	10.522 ± 0.807	0.554 ± 0.057	3.490 ± 0.337
TPPF-BS-0.25%	0.471 ± 0.231	15.119 ± 1.015	0.344 ± 0.156	2.125 ± 0.681
TPPF-BS-0.50%	0.520 ± 0.167	14.213 ± 0.648	0.397 ± 0.107	2.344 ± 0.539
TPPF-BS-0.75%	0.568 ± 0.103	13.307 ± 0.283	0.450 ± 0.058	2.563 ± 0.398
TPPF-BS-1.00%	0.617 ± 0.046	12.402 ± 0.103	0.502 ± 0.014	2.782 ± 0.262
TPPF-BS-1.25%	0.618 ± 0.011	14.209 ± 2.382	0.511 ± 0.015	2.868 ± 0.211
TPPF-BS-1.50%	0.620 ± 0.067	16.016 ± 4.724	0.520 ± 0.043	2.955 ± 0.593

**Table 7:** Screening basis for candidate reinforcement schemes used in the true triaxial tests.

Candidate Group	Main Screening Basis	Selection Purpose
PVA-1.00%	Good tensile reinforcement, deformation coordination, and relatively stable buffered shear response.	Candidate representing tensile–deformation coordination.
TPPF-0.75%	Highest shear cohesion in the variable-angle shear test and good load-bearing response under buffered shear loading.	Candidate representing shear-resistance reinforcement.

## Data Availability

The original contributions presented in this study are included in the article. Further inquiries can be directed to the corresponding author.
